# Metabolomics Technologies for the Identification and Quantification of Dietary Phenolic Compound Metabolites: An Overview

**DOI:** 10.3390/antiox10060846

**Published:** 2021-05-25

**Authors:** Anallely López-Yerena, Inés Domínguez-López, Anna Vallverdú-Queralt, Maria Pérez, Olga Jáuregui, Elvira Escribano-Ferrer, Rosa M. Lamuela-Raventós

**Affiliations:** 1 Department of Nutrition, Food Science and Gastronomy XaRTA, Institute of Nutrition and Food Safety (INSA-UB), Faculty of Pharmacy and Food Sciences, University of Barcelona, 08028 Barcelona, Spain; naye.yerena@gmail.com (A.L.-Y.); idominguez@ub.edu (I.D.-L.); avallverdu@ub.edu (A.V.-Q.); mariaperez@ub.edu (M.P.); 2 CIBER Physiopathology of Obesity and Nutrition (CIBEROBN), Institute of Health Carlos III, 28029 Madrid, Spain; 3 Laboratory of Organic Chemistry, Faculty of Pharmacy and Food Sciences, University of Barcelona, 08028 Barcelona, Spain; 4 Scientific and Technological Center (CCiTUB), University of Barcelona, 08028 Barcelona, Spain; ojauregui@ccit.ub.edu; 5 CIBER Fragilidad y Envejecimiento Saludable (CIBERfes), Instituto de Salud Carlos III, 28029 Madrid, Spain; 6 Biopharmaceutics and Pharmacokinetics Unit, Department of Pharmacy and Pharmaceutical Technology and Physical Chemistry, Institute of Nanoscience and Nanotechnology (IN2UB), Faculty of Pharmacy and Food Sciences, University of Barcelona, 08028 Barcelona, Spain; 7 Pharmaceutical Nanotechnology Group I+D+I Associated Unit to CSIC, University of Barcelona, 08028 Barcelona, Spain

**Keywords:** phenolic compounds, metabolites, sample treatment, NMR, LC/MS, urine, plasma

## Abstract

In the search for natural products with properties that may protect against or slow down chronic and degenerative diseases (e.g., cancer, and cardiovascular and neurodegenerative conditions), phenolic compounds (PC) with benefits for human health have been identified. The biological effects of PC in vivo depend on their bioavailability, intestinal absorption, metabolism, and interaction with target tissues. The identification of phenolic compounds metabolites (PCM), in biological samples, after food ingestion rich in PC is a first step to understand the overall effect on human health. However, their wide range of physicochemical properties, levels of abundance, and lack of reference standards, renders its identification and quantification a challenging task for existing analytical platforms. The most frequent approaches to metabolomics analysis combine mass spectrometry and NMR, parallel technologies that provide an overview of the metabolome and high-power compound elucidation. In this scenario, the aim of this review is to summarize the pre-analytical separation processes for plasma and urine samples and the technologies applied in quantitative and qualitative analysis of PCM. Additionally, a comparison of targeted and non-targeted approaches is presented, not available in previous reviews, which may be useful for future metabolomics studies of PCM.

## 1. Introduction

For several years, the benefits of phenolic compound (PC) intake have been described, especially as a protection from cardiovascular diseases [1,2]. The biological effects of PC in vivo depend on their bioavailability, intestinal absorption, metabolism, and interaction with target tissues [3]. It is well-known that PC have a low oral bioavailability, and undergo an extensive biotransformation mediated by phase I reactions (e.g., oxidation, reduction, or hydrolysis), phase II reactions (e.g., glucuronidation, methylation, sulfation), as well as by gut microbiota (e.g., ring fission, hydrolysis, demethylation, reduction, decarboxylation, dihydroxylation, and isomerization) [4,5]. In fact, the identification, in biological samples, of the phenolic compounds’ metabolites (PCM) produced after foods rich in PC intake, is a first step to understand the overall effect on human health [6]. Unfortunately, the (i) enormous structural variety of PC (i.e., phenolic acids, flavan-3-ols, flavanones, flavones, flavonoids, lignans, among others), (ii) the lack of commercially available standards, (iii) the structural variability of PCM, (iv) metabolite degradation due to tedious multi-step sample preparation workflows, (v) instability of specific compounds, (vi) low analyte concentration, and (vii) frequent sample contamination [7,8,9,10], render their identification a complex task. In the last decades, pre-analytical separation processes and more powerful separation systems for their analysis have been applied.

Accurate identification and quantification of analytes greatly depends on the extraction step [7,8,9,10,11]. Sample preparation, also known as sample pretreatment or clean-up, is a mandatory step to ensure adequate sensitivity, selectivity, and reproducibility of the analytical process in metabolomics analyses. The objectives of the pre-analytical separation process include: (i) minimization or elimination of interferences or undesired endogenous compounds in the extracted samples, (ii) enhanced selectivity for targeted analytes, (iii) sample preconcentration to improve assay sensitivity, and (iv) sample stabilization by reconstituting the mixture in an inert solvent. The development of analytical methods for biological samples has become increasingly challenging due to growing demands for method reliability and sensitivity, and speed of analysis and sample throughput. However, the extraction approach used is often dictated by the characteristics of the target analyte (hydrophilicity, lipophilicity, and protolytic properties) and the matrix. In liquid chromatography-mass spectrometry (LC-MS)-based metabolite analyses, sample preparation depends on whether a targeted or non-targeted strategy is employed [11]. In non-targeted analysis, the main methods of sample clean-up are the “dilute and shoot” approach, protein precipitation (PP), and ultrafiltration [11]. In targeted analysis, liquid-liquid extraction (LLE), solid-phase extraction (SPE), solid-phase microextraction (µSPE), solid-liquid extraction (SLE), and PP are the most commonly used [11].

In the SPE technique, analytes from the sample solution or extract are concentrated and purified by sorption on a solid sorbent [12]. SPE efficiency depends on the nature of the stationary phase, solvent and volume sample, solvent pH, and modifier content [13]. An alternative to conventional SPE, µSPE allows analyte extraction from a small amount of sample using a low elution volume. Its advantage is that sample extraction and preconcentration can be performed in one step, avoiding evaporation and reconstitution, and therefore reducing the time of analysis [14]. LLE is a technique used to isolate target compounds that consists of adding an immiscible organic solvent, commonly ethyl acetate, to the biological sample, which is subjected to vortex-mixing and centrifugation to achieve the transfer of the analytes to the organic phase [7]. This technique stands out for being simple to assemble and providing pure extracts, resulting in improved sensitivity in the chromatographic column [15]. However, if the number of samples is large, LLE is a slow procedure and requires a high consumption of solvents [16]. Finally, PP involves treating the samples with a protein precipitant (e.g., water-miscible organic solvents such as acetonitrile (ACN), acetone, ethanol, and methanol), its efficiency depending on the volume of organic solvent added and the presence of acid [7].

The analytical techniques commonly used for compound identification can measure a wide range of molecule classes, but each has its limitations regarding detection, sensitivity, dynamic range, and quantification. In metabolomics, the analytical tools of choice for small-molecule analysis are mass spectrometry (MS) and nuclear magnetic resonance (NMR) [17]. Chromatographic techniques for the separation of target compounds, typically coupled with MS, are liquid chromatography (LC), gas chromatography (GC), or capillary electrophoresis (CE) [7,17,18].

MS is a highly sensitive method that can detect, quantitate, and structurally elucidate several hundred metabolites in a single measurement [17,18]. The sensitivity and accuracy of detection by MS depend on the experimental conditions and instrumental settings, especially the methods of metabolite extraction, separation, and ionization (and possibly ion suppression) [17]. The most commonly applied ionization techniques, which are key for compound detection and quantification [17], are electrospray ionization (ESI), atmospheric pressure chemical ionization (APCI), and matrix-assisted laser desorption ionization (MALDI) [11,18,19]. The ionization charges neutral molecules so they can be manipulated by electrical and/or magnetic and/or radio frequency energies according to their m/z ratio [19]. The most conventional method of ion-fragmentation is collision-induced dissociation (CID), although surface-induced dissociation (SID) and infrared multiphoton dissociation (IRMPD) have also been employed [18]. Target compound detection with a high degree of resolution and sensitivity generally cannot be achieved using a single MS detection mode, as higher sensitivity leads to lower resolution, and vice versa [17]. Single-configuration mass analyzers include quadrupole (Q), linear ion trap (LIT), quadrupole ion trap (QIT), time of flight (TOF), Fourier transform ion cyclotron resonance (FTICR), and Orbitrap. Hybrid configuration can further aid metabolite identification by providing highly resolved and accurate MS/MS spectra [20]. Tandem (MS/MS) mass analyzers for quantitative and qualitative metabolomics studies include triple-quadrupole ion trap (QTrap), triple quadrupole (QqQ), quadrupole-time-of-flight (Q-TOF), and linear-quadrupole ion trap-Orbitrap (LTQ-Orbitrap) [17,18,20].

The aim of this review is to highlight recent advances in PCM identification and quantification in plasma and urine from humans and animals after the consumption of dietary PC. Sample preparation methods that enhance analytical sensitivity, identification, confidence, or the range of PCM that can be identified in these biological samples are described. Finally, the limitations and advantages of analytical methods in metabolomics studies are outlined.

## 2. Biological Sample Preparation Methods

**Figure 1:**
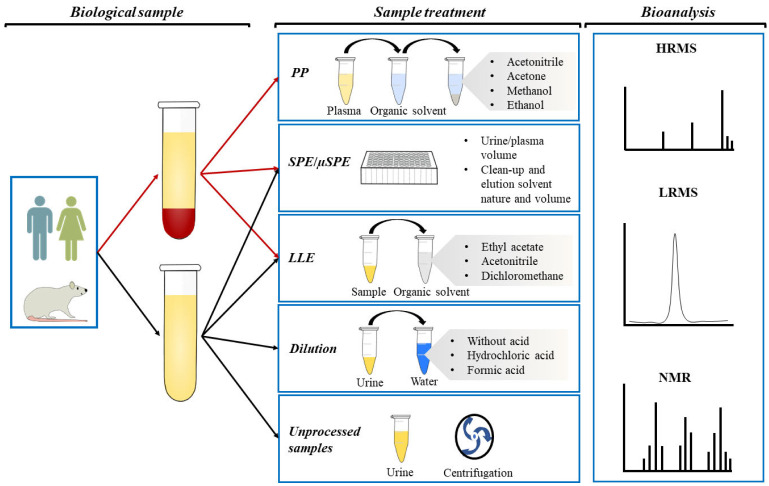
Graphical representation of plasma and urine clean-up and the bioanalysis for the identification and quantification of phenolic metabolites.

There has been considerable progress in the development of sample preparation methods that allow the detection of PC and PCM with differentiated physicochemical properties. In LC-MS-based metabolite analyses, the main sample preparation methods used in non-targeted strategies are “dilute and shoot”, PP, and ultrafiltration, whereas LLE, SPE, µSPE, SLE, and PP are the most common techniques in targeted analysis [11]. Table 1, drawing on work of the last two decades, includes the dietary source of each analyzed PC, the precursor compounds, the PCM type, and the biological sample analyzed. Additionally, detailed information about PCM of secoiridoids, phenolic alcohols, flavonoids, phenolic acids, enterolignans, stilbenes, ellagitannins, and others is presented in the Supporting Information (Supporting Information (Tables S1, S2, S3, S4, S5, and S6, respectively). Figure 1 shows the general clean-up steps for human or animal plasma and urine.

### 2.1. Plasma

Plasma is an aqueous solution that contains a great variety of substances, including proteins, glucose, mineral ions, hormones, carbon dioxide, and blood cells [8], some of which may interfere with detection of the target analyte. As PCM may have a higher binding affinity to plasma proteins than the parent compounds [99], proteins and other cellular components such as phospholipids need to be removed prior to analysis to reduce potential matrix effects and ion suppression phenomena [100]. An appropriate sample treatment is therefore a crucial step that influences the reliability and accuracy of the analysis [16].

#### 2.1.1. Solid-Phase Extraction

PC and PCM extraction from plasma samples is typically performed by C18-bonded SPE, as it produces results with greater repeatability than techniques such as PP [101]. Other advantages over methods such as LLE include higher recoveries, shorter analysis time, and lower solvent requirements [12].

A range of solvents have been used to extract anthocyanins and their metabolites from plasma by SPE. PCM elution is frequently carried out with methanol, which efficiently precipitates proteins and deactivates enzymes, and can be combined with water to improve the extraction of polar phenolic acids. Some studies have used methanol acidified with different percentages of formic acid to elute anthocyanins and their metabolites in C18 cartridges [55]. The isolation of anthocyanins and their glucuronide metabolites was also achieved using hydrophilic-lipophilic-balance (HLB) cartridges and eluting the analytes with acetone/formic acid (9:1), obtaining a recovery rate of 71% for cyanidine-3-glucose [51]. Anthocyanin and phenolic acid metabolites were also eluted with acetone/formic acid (9:1) [56]. Acetone has an advantage over methanol in that its lower boiling point allows a faster evaporation.

The extraction of different classes of flavonoids and their metabolites was performed using methanol as the elution solvent in mixed-mode cation-exchange (MCX) plates [82]. The samples were previously hydrolyzed or nonhydrolyzed to analyze the metabolites in both their free and conjugated forms. With this procedure, phase-II metabolites of flavonoids and their microbial derivatives were identified and quantified, with recovery values ranging from 87% to 109%. Epicatechin and its metabolites were extracted with different solvents using Strata X cartridges. Actis-Goretta et al., employed methanol/pyridine (1:1 *v/v*) to elute the metabolites, mainly glucuronides, sulfates, and *O*-methyl sulfates, achieving recoveries from 96% to 104% [77]. These results constitute an improvement on previous studies that used HLB cartridges with methanol containing formic acid (0.1%) [76] or *N,N*-dimethyl formamide/methanol (7:3).

The first validated methodology for the quantification of oleuropein and its metabolites in plasma was based on sample pretreatment with SPE, using HLB cartridges and acetone as the eluent. This approach allowed the simultaneous determination of metabolites previously undetected by other techniques such as LLE, namely 2-(3,4-dihydroxyphenyl)acetic acid, homovanillyl alcohol, homovanillic acid, and elenolic acid [21].

In a study on procyanidin metabolites, rat plasma samples after ingestion of procyanidin-enriched cocoa cream were pretreated in offline SPE HLB cartridges. The retained compounds were eluted with acetone/water/acetic acid (70:29.5:0.5, *v/v/v*), which produced better results than methanol/acetone, with the recoveries being over 84%. The isolated procyanidin metabolites were mainly phase-II-conjugated catechin-glucuronide and epicatechin-glucuronide [73].

A method based on high-throughput µSPE using HLB µElution plates was developed by Feliciano et al. for the identification and quantification of different classes of PCM, including flavonols, benzoic acids, catechols, and cinnamic acids. Recovery rates of 89% were obtained when the µ-elution was performed with methanol followed by 70% aqueous acidified acetone [57].

Suárez et al. obtained the highest recovery of hydroxytyrosol using 50% aqueous ACN, although this method was less efficient for other PCM, whose recoveries were better with methanol [14]. The µSPE method has been used for the analysis of hydroxytyrosol and derivatives in plasma samples [26,31]. In another study evaluating methanol as the elution solvent, recoveries of over 75% were achieved for all the studied PCM, including catechol sulfate and valerolactone glucuronide [67].

#### 2.1.2. Liquid-Liquid Extraction

In a study on the pharmacokinetic processes of flavan-3-ols [96], LLE was carried out to extract phase-II-conjugated compounds and gut microbiota metabolites. The spiked samples, previously acidified with formic acid, were extracted 3 times with ethyl acetate, yielding recoveries of 70–100%. However, the values for cinnamic acids varied considerably, suggesting that the solvent was not suitable for its extraction. Ethyl acetate was also employed in an LLE of gallic acid metabolites in human plasma, with the recoveries ranging from 67% to 96% [89], and in the clean-up of rat plasma containing caffeic, ferulic, and isoferulic acid metabolites [91].

#### 2.1.3. Protein Precipitation

Plasma contains a large amount of proteins that should be removed to avoid interference in the final analysis [99]. Samples are treated with a protein precipitant, commonly water-miscible organic solvents such as ACN, acetone, perchloric acid, ethanol, and methanol. Both composition and volume of the organic precipitant can affect PP efficiency, which may be improved by adding an acid (e.g., formic, acetic, trichloroacetic, and phosphoric) to the sample or PP solution to alter the pH of the sample matrix [7]. Although PP is a simple and fast method, it does not provide a completely clean extract. Protein removal can also be achieved by the addition of acids such as trichloroacetic or perchloric acids, which have a denaturation and aggregation effect.

Other components of plasma samples that could interfere with the analysis are phospholipid species, which may impair both the ionization process and chromatographic separation, thereby reducing extraction efficiency, recovery, and reproducibility, and increasing inter-sample variability. The removal of phospholipids (highly ionic) has been successfully accomplished with recently developed commercial plates [102].

ACN and formic acid (2%) were used to extract red raspberry anthocyanins and ellagitannins and their phase-II metabolites from human plasma [53]. The same solvents were employed in an analysis of phase-II metabolites of flavonoids and phenolic acids and phase-II and microbial metabolites of flavanone in plasma after the consumption of orange juice.

Ethanol has been utilized in different studies as a protein precipitant, including in an evaluation of chlorogenic and phenolic acids and their metabolites in human plasma after coffee consumption, and the recoveries ranged from 98.1% to 108.6% for all the glucuronide, sulfate, and lactone metabolites of the studied phenolic acids [36]. A similar procedure was performed to isolate chlorogenic acids and their metabolites, although the plasma samples were resuspended in sodium acetate buffer and acidified with hydrochloric acid in aqueous methanol (40%, *v/v*), achieving an 88% recovery of chlorogenic acid [35]. In another study, coffee phenolic metabolites thought to be formed by the gut microbiota were identified after ethanol-induced PP and sample acidification with perchloric acid [37].

### 2.2. Urine

Urine contains high concentrations of urea, inorganic salts (chloride, sodium, and potassium), creatinine, ammonia, organic acids, various water-soluble toxins, and pigmented products of hemoglobin breakdown [103]. Although urine is one of the easiest biological fluids to collect, its high salt concentration can easily interfere with ESI. Thus, the electrolytes need to be removed prior to the analysis [27], which is usually partially achieved by SPE, LLE, and sample dilution. Nevertheless, direct analysis of urine (unprocessed urine) has also been carried out.

#### 2.2.1. Solid-Phase Extraction

One of the most widely used techniques for the preconcentration and clean-up of urine samples is SPE, in which the nature and volume of the elution solvent are important factors [73]. This technique has been used for metabolite extraction of PC from the main dietary sources such as tomato [3], tomato sauce [80], extra virgin olive oil [22,25,30], red wine [49,104], black raspberry [52], chokeberry [54], cocoa [70,71,79], chocolate [77], and anthocyanins [105]. An SPE method for the quantification of 350 dietary markers, including (poly)phenolic aglycones, phase-II metabolites, microbial-transformed compounds, and other dietary components, was also developed [106].

Methanol and water are by far the most extensively used solvents for PCM extraction [3,22,25,30,52,54,70,71,77,79,80,105]. Methanol and ethyl acetate have also been used to isolate PCM, specificity, and resveratrol metabolites [49,104]. On the other hand, the methanol and ammonium formate have been less used [106].

Another strategy to optimize the PCM extractions is the use of different additives during its washing and elution. In this sense, formic acid [3,30,52,70,71,77,80,105,106], acetic acid [22,49,104], and trifluoroacetic acid [54] are the most common additives used. In other studies, both the methanol and water were employed without the addition of any additive [25,79].

In some cases, urine dilution, before SPE extraction, may be necessary to reduce the ionic strength prior to ion-exchange SPE due to a high concentration of salts [7]. Water [3], water with trifluoroacetic acid [22,54], water with phosphoric acid [25,49,104], water with formic acid [106], and methanol [30] have been extensively used prior to SPE extraction. In other studies, the urine dilution was not employed [52,70,71,79]. Finally, in another study, it was demonstrated that a complete evaporation of the eluent (to dryness) significantly reduced the recovery of certain compounds [105].

µSPE constitutes an excellent option for PCM analysis as it requires small sample volumes. Feliciano and coworkers developed a rapid and high-throughput µSPE method for the analysis of the most representative polyphenol classes (flavan-3-ols, benzoic acids, phenylacetic acids, propionic acids), achieving recoveries of up to 88% when using methanol followed by 70% aqueous-acidified acetone as the elution solvent [57]. In 2013, Serra demonstrated that a clean-up step was not necessary in the µSPE method for hydroxytyrosol metabolites, which was attributed to their polarity. Thus, these compounds were eluted with water without affecting the matrix effect (lower than 18%) [24]. µSPE was used with the aim to detect phenol metabolites in urine samples to identify the most appropriate compliance olive oil markers and eventually relate them to the expected biological effects [31].

#### 2.2.2. Liquid-Liquid Extraction

This technique has been used for the extraction of PCM from the main dietary sources, such as extra virgin olive oil [27,107], orange juice [44], tea [38], yerba mate [42], puree of five (poly)phenol-rich berry fruits [66], cocoa products [72], and specific compounds, such as resveratrol [90] and daidzein and genistein [78].

ACN has been extensively employed for LLE of PCM [44,46,48,66]. In other studies, ethyl acetate [27,107] and dichloromethane have also been employed for the extraction of PCM [38]. Although LLE can be carried out with a single solvent, the use of solvent mixtures can improve extraction [7]. An ACN/methanol mixture (8:2, *v/v*) was used to extract major phase-II metabolites of resveratrol [90], adding an acetate buffer to increase the density of the aqueous layer.

LLE constitutes an excellent option for PCM analysis as it requires small urine volumes (<500 µL). Another advantage of LLE is that sample extraction can be performed in one step, avoiding evaporation and reconstitution, and therefore reducing the time of analysis. In this sense, after centrifugation (and/or filtration), the samples can be directly injected in MS.

#### 2.2.3. Urine Dilution

The preparation of samples using the “dilute and shoot” approach reduces the time required to obtain results [11]. Different volumes of water (1:1, 1:3, 1:4, 1:5, or 1:10 *v/v*, urine:water), with or without formic acid (0.1%) or acetic acid [23], have been used to analyze a wide variety of PCM. After dilution, the samples are usually mixed, centrifuged, and filtered prior to the bioanalysis. In 2019, González-Domínguez and colleagues demonstrated that a 10-fold dilution factor was optimum for a minimal matrix effect provoked by the high salt content of urine [106]. The same authors found that this strategy yielded LOQs above 10 μg L^−1^ for almost all compounds monitored, which can hinder the detection of food-derived metabolites in real urine samples. Hydrochloric acid (0.1 M) has been employed in urine treatment for the analysis of microbial metabolites, glucuronides, and sulfate derivatives [64]. The same acid was used to retard bacterial growth and ensure metabolite stability in human urine after the consumption of black tea [64].

#### 2.2.4. Unprocessed Urine

The direct injection of urine samples is known to be difficult due to problems such as column clogging and MS signal alterations caused by endogenous compounds. Nevertheless, unprocessed urine samples have been analyzed, in nontargeted approaches, to determine the profile of excretable PC. Unprocessed urine containing metabolites of flavonoids and hydroxycinnamic acids after the acute consumption of grape juice [62], hydroxycinnamate derivatives after the acute consumption of coffee [34], and PCM after polyphenol-rich juice drink [65] and moderate wine [48] consumption have been analyzed. The urine samples were centrifuged, to eliminate cells and noncellular suspended particles, before being directly analyzed by UHPLC−TOF-MS [48] or HPLC-PDA-LIT [34,62,65].

## 3. Identification and Quantitation of Metabolites

### 3.1. Nontargeted Approaches

Nontargeted approaches to PC and PCM characterization are becoming more feasible with the high analytical power provided by high-resolution mass spectrometry (HRMS) instruments (MS and MS/MS mode) [108,109]. In Table 2, the main chromatographic and MS conditions employed in nontargeted studies of PCM in urine and plasma samples are summarized.

Q-TOF-MS is a hybrid analytical technique widely used in metabolomics. This combination of analyzers takes advantage of the higher MS/MS efficiency demonstrated by QqQ instruments and the speed and sensitivity of a TOF analyzer [69,110,111]. A Q-TOF-based metabolomics approach is a suitable strategy to understand the effect of nutritional interventions, reveal metabolomic changes, and obtain data on bioaccessibility and bioavailability [111]. A comprehensive list of the main conditions used to analyze dietary PCM in urine and plasma is provided in Table 2.

Although Q-TOF-MS has been applied to detect phase-I metabolites in vivo [60,86], conjugated metabolites have received more attention. Thus, glucuronide and sulfate forms of quercetin were the main metabolites identified in human plasma post-consumption of applesauce enriched with apple peel and onion [69]. In another study, phase-II metabolites were identified in rat plasma and urine after a single oral administration of quercetin [86]. Human urinary metabolites, mainly sulfated forms of caffeic and ferulic/isoferulic acids, were detected after consumption of yerba mate [42]. Other studies have focused on the identification of PCM after ingestion of cranberry syrup [60], cranberry juice [57], orange juice [44], and chocolate [79].

The key role played by phenolic microbial metabolites in the health effects of dietary PC has prompted experiments on Q-TOF-based metabolomic fingerprints in biological fluids. Gut microbial metabolites were analyzed in plasma and urine samples following the consumption of cranberry juice [57], cocoa products [72], beans [83], and tomato products [81]. Hydroxycinnamate metabolites derived from microbiota were detected in both plasma and urine after coffee intake [32], and microbial metabolites in urine were found to be strongly affected by moderate red wine consumption [48]. In summary, negative ESI (Table 2) is the most frequently used mode in analysis of PCM, regardless of whether they are phase I, phase II, or microbial metabolites. Regarding chromatographic separation, the most employed mobile phases are water and ACN with formic acid (0.1% or 0.01%).

The prediction, screening, and identification of PCM in foods is rarely straightforward. The Orbitrap mass analyzer, the first high-performance mass analyzer able to trap ions in electrostatic fields, provides the high-resolution, mass accuracy, and sensitivity required for metabolomic analyses [112,113]. Two major families of instruments are used to study PCM: the hybrid linear ion-trap Orbitrap MS (LTQ-Orbitrap) and Q Exactive. Table 2 summarizes the chromatographic and MS conditions used in the analysis of PCM.

In the most frequently employed operation mode, the Orbitrap mass analyzer acquires FTMS data and the LTQ provides data-dependent MS/MS scans after trapping. The advantages are the high trapping capacity and MS^n^ scanning function of the LTQ along with accurate mass measurements, resulting in greater throughput and identification of major or trace metabolites. To date, the LTQ-Orbitrap mass analyzer is the most popular platform used in dietary PC metabolomics research, applied to study the metabolism of compounds such as (-)-epicatechin [87], oleocanthal [93,94], oleacein [95], daidzein [88], hesperetin, and hesperidin [92], and to identify PCM in biological samples after the intake of foods or beverages [41,64,66,81,84]. As can be seen in Table 2, negative ESI is the most routinely used mode in PCM analysis. Although high mass resolution is necessary to separate or resolve two peaks with a small mass difference, in most of the studies, the resolution was set at 30,000 in FTMS mode. A resolution of 60,000 was used in only one study and 70,000 in another. Regarding the chromatographic conditions, the most employed mobile phases are water and ACN or methanol with formic acid (0.1% or 0.01%).

Another newly developed method is Q Exactive hybrid quadrupole-orbitrap mass spectrometry (Q Exactive MS), which combines high-performance quadrupole precursor selection with high-resolution and accurate-mass orbitrap detection. In the field of PC analysis, from 2015 until the present, this hybrid technology has been used in only a few studies, to identify proanthocyanidin [50], procyanidins [59], resveratrol [106], flavanone, and secoiridoids [29]. Most (80%) of these studies used heated electrospray ionization (HESI), which generally gives improved signals compared to the more traditional unheated ESI (Table 2). Chromatographic separation was carried out as in the LTQ-Orbitrap system, using water and ACN or methanol with formic acid (0.1% or 0.01%).

### 3.2. Targeted Approaches

Targeted analyses require the collection of specific metabolite information, typically through the use of low-resolution mass spectrometry (LRMS) instrumentation. There are several types of mass analyzers that can generate mass measurements, but the most frequently used are QqQ or QTrap [114]. Table 3 summarizes the main chromatographic and MS conditions employed in targeted studies of PCM in urine and plasma samples. It is well-known that due to the lack of bioanalytical standards for some PCM, various strategies have been used trying to solve this problem: (i) quantification of PCM using the calibration curves corresponding to their phenolic precursors or with the most structurally similar compound [42,43], (ii) expressing the concentration as equivalents of parent compounds [71,94], (iii) expressing the concentration as ratios [29,93,95,115], and (iv) the synthesis of the target metabolites [39]. In the case of the expression of the concentration as ratios, the peak abundance ratio (analyte/internal standard) and the concentration ratio (analyte/internal standard) are used [115]. In other cases, the peak area ratios of each analyte vs. that of the internal standard (analyte/internal standard) are employed [21,63].

#### 3.2.1. Quadrupole Ion Trap (QIT)

QIT, despite being one of the first technologies employed to analyze dietary PC, has been applied in only a few studies to date. Its principal limitation is a relatively low limiting value of the mass/charge ratio. As can be seen in Table 3, the quantification of PCM with this technology has focused on flavonoids [38,62,65,116] and phenolic acids [62], both in plasma and urine samples, using ESI and fragmentation by CID. A single mobile phase was employed in two studies [62,65], whereas others used a combination of two [116] or three [38]. In all cases, formic or acetic acids (0.05%, 0.1%, or 0.5%) were the major additives used to improve compound ionization.

#### 3.2.2. Triple Quadrupole (QqQ)

Due to its high sensitivity and selectivity, QqQ mass spectrometry has been primarily applied in targeted analysis of dietary PCM. Table 3 summarizes the chromatographic and MS conditions routinely used in the study of PCM.

Phase-I metabolites of oleuropein in plasma samples were analyzed with QqQ technology, employing a C8 column with water (pH 5, adjusted with acetic acid) and ACN for chromatographic separation [21]. To quantify phase-I and phase-II metabolites of oleocanthal, a C18 column and water:MeOH, both with 0.1% formic acid, was used [93]. In addition, phase-II metabolites of phenolic acids [14,36], phenolic alcohols [14,25], flavonoids [14,77], phenyl-γ-valerolactones [39], procyanidins [117], and resveratrol [90] were studied, employing a C18 column and water:ACN for plasma samples and ammonium acetate:ACN for urine.

Water and ACN containing formic acid (0.1%, 0.2%, and 0.5%) in combination with C18 columns have been extensively used in the analysis of phase-II metabolites and microbial metabolites of flavonoids [56,61,66,68,71,80] and phenolic acids [61,66,80] in plasma or urine samples. The same mobile phases have been used in the analysis of epicatechin and procyanidin microbial metabolites [70] in urine samples. In another study, a PFP column was used to separate polar compounds, including phenolic acid metabolites [53].

#### 3.2.3. Triple-Quadrupole Ion Trap (QTrap)

QTrap is the one of the most widely used instruments for analyzing specific metabolites of interest [108]. Recently, targeted metabolomic profiling of the urinary [106] and plasma [118] food metabolome has resulted in the simultaneous quantitation of PCM, including (poly)phenolic aglycones, conjugated metabolites, and colonic microbial metabolites. QTrap technology has been extensively utilized to develop a targeted metabolomic approach for specific PC groups, being applied to plasma and human urine containing phenolic acid metabolites [119] or specific compounds such as chlorogenic acid [33]. Regarding flavonoids, phase-I, phase-II, or microbial metabolites were detected in plasma [97] and urine [46,78,105]. As shown in Table 3, acetic and formic acids are the major additives used to improve compound ionization. The most common mobile phases for urine are both ammonium acetate and water (with acid), together with ACN/methanol, and for plasma, water, and ACN.

**Table 3:** Chromatographic and MS conditions for the quantification of PCM in plasma and urine samples.

Phenolic Compounds	Sample; Sample Clean-Up; Internal Standard	Chromatographic Conditions	MS Conditions	LOQ
*Phase I Metabolites*				
Oleuropein [21]	Plasma; SPE; 2-hydroxyphenylethanol	RP-C8 (150 × 2.1 mm, 3.5 μm); A: Water pH 5, adjusted with AA. B: ACN	QqQ; MRM; ESI-[M−H]^−^	5–50 ng L^−1^
*Phase-II Metabolites*				
Flavonoids and phenolic acids [65]	Plasma and urine; PP and UU; Ethyl gallate	Synergi RP-Max (4.6 × 250 mm, 4 μm); MeOH (0.5% AA or 1% FA).	QIT; FS, CRM, and SIM; ESI- [M−H]^−^/ [M+H]^+^	
Flavonoids [116]	Plasma and urine; LLE, SPE, and PP	Symmetry C18 (2.1 × 50 mm, 3.5 μm); A: Water, B: ACN, both with 0.05% FA.	QIT; FS; ESI- [M−H]^−^/ [M+H]^+^	
Catechins [38]	Urine; LLE	Supelcosil LC18 (3.0 × 100 mm, 5 μm); A: MeOH-water-AA (1:98:1), B: MeOH- water-AA (50:49:1), C: MeOH-water (4:1).	QIT; FS; ESI- [M−H]^−^	
Phenolic acids [36]	Plasma; PP; mixture solution *	Acquity BEH C18 (2.1 × 150 mm, 1.7 μm); A: Water, B: ACN, both with 1% AA.	QqQ; MRM; ESI- [M−H]^−^	5 nM
Procyanidins [117]	Plasma; SPE; Catechol	Acquity HSS T3 (2.1 × 100 mm, 1.8 μm); A: Water (0.2% AA), B: ACN.	QqQ; SRM; ESI- [M−H]^−^	0.01–0.98 µM
Phenolic acids and alcohols and flavonoids [14]	Plasma; µSPE; Catechol and caffeic acid	Acquity BEH C18 (2.1 × 100 mm, 1.7 µm); A: Water (0.2% AA), B: ACN.	QqQ; SRM; ESI- [M−H]^−^	0.02–8.2 µM
Phenolic alcohols [25]	Urine; SPE; 4’-*O*-hydroxy phenylpropanol and 3-(4- hydroxyphenyl) propanol	Acquity BEH C18 (2.1 × 100 mm, 1.7 µm); A: 1 mM ammonium acetate (pH 5), B: ACN	QqQ; MRM; ESI- [M−H]^−^	1.87–20 ng L^−1^
Resveratrol [90]	Urine; UD; Wogonin	ODS-3 (5 × 150 mm, 2.1 μm); A: 5 mM ammonium acetate, B: ACN	QqQ; SRM; ESI- [M−H]^−^	4–20 ng L^−1^
(─)-Epicatechin [77]	Plasma and urine; SPE; Umbelliferone sulfate and glucuronide	HSS C18 (1.8 × 100 mm, 2.1 μm); A: Water, B: ACN, both with 0.1% AA.	QqQ; MRM; ESI- [M−H]^−^	12–30 nM;
Phenyl-γ-valerolactone [39]	Plasma and urine; PP and UD	Kinetex EVO C18 (2.1 × 100 mm, 2.6 μm); A: Water, B: ACN, both with 0.2% FA.	QqQ; MRM; HESI- [M−H]^−^	0.6–2.2 nM
Flavonoids and phenolic acids [43]				
Daidzein and genistein [78]	Urine; LLE; Taxifolin	Phenomenex C18 (3 × 150 mm, 3 μm); A: ammonium acetate (13 mM, pH 4 with 0.1% AA), B: MeOH (0.1% AA).	QTRAP; MRM; ESI- [M−H]^−^	3 ng mL^−1^
Luteolin [97]	Plasma; PP	Capcell pak C18 MGII (4.6 × 150 mm, 3 μm); A: water (0.1% TFA), B: ACN.	QTRAP; MRM; ESI- [M+H]^+^	
*Phase I and II Metabolites*				
Chlorogenic acid [33]	Urine and plasma; PP; mixture solution *	Synergi Polar RP C18 (4.6 × 250 mm, 4 µm); A: Water, B: ACN, both with 0.1% AA.	QTRAP; MRM; ESI- [M−H]^−^	5 nM
Flavonoids and phenolic acids [62]	Plasma and urine; SPE and UU; Cyanidin-3-*O*-sambubioside-5-*O*-glucoside	Synergi Polar RP C18 (4.6 × 250 mm, 4 µm); MeOH (0.5% AA or 1% FA).	QIT; FS, SRM, SIM, and CRM; ESI-[M−H]^−^/ [M+H]^+^	
Oleocanthal [93]	Plasma; SPE	AcQuity BEH C18 (2.1 × 50 mm, 1.7 µm); A: Water, B: MeOH, both with 0.1% FA.	QqQ; MRM; ESI- [M−H]^−^	
Anthocyanins [120]	Plasma and urine; SPE; Scopoletin	Kinetex PFP (4.6 × 100 mm, 2.6 μm); A: Water, B: ACN, both with 0.1% FA.	QTRAP; MRM; ESI- [M+H]^+^	
*Microbial Metabolites*				
Epicatechin, procyanidins [70]	Urine; SPE; Ethyl gallate	Luna C18 (2.0 × 50 mm, 5 μm); A: Water/ACN (94.9:5, *v/v*), B (ACN), both with 0.1% FA.	QqQ; MRM; ESI- [M−H]^−^	0.03–44.4 μg L^−1^
Flavanones [46]	Urine; LLE	ACE 3 C18-AR (4.6 × 75 mm, 3 μm); A: Water, B: ACN, both with 0.1% FA.		
*Phase-II and Microbial Metabolites*				
Flavonoids and phenolic acids [66]	Urine; LLE; Rutin and taxifolin	Atlantis T3 (2.1 × 100 mm, 3 µm); A: Water/ACN (95:5, *v/v*), B: ACN/water (95:5, *v/v*), both with 0.5% FA.	QqQ; MRM; ESI- [M−H]^−^	
(─)-Epicatechin [71]	Plasma and urine; SPE; Ethyl gallate	Luna C18 (2.0 × 50 mm, 5 µm); A: Water, B: ACN, both with 0.1% FA.	QqQ; MRM; TIS- [M−H]^−^	
Flavonoids and phenolic acids [80]			QqQ; MRM; ESI- [M−H]^−^	28.4–75.8 ng L^−1^
Flavonoids and phenolic acids [61]	Plasma and urine; SPE and UD	Kinetex EVO C18 (2.1 × 100 mm, 2.6 μm); A: Water, B: ACN, both with 0.2% FA.	QqQ; SRM; HESI- [M−H]−	
Flavan-3-ols [68]	Urine; UD		QqQ; MRM; ESI- M−H]^−^	
Phenolic acids [56]	Plasma; SPE; Syringic acid	Pursuit 3 PFP (150 × 2.0 mm); A: Water, B: ACN, both with 0.1% FA.	QqQ; MRM; ESI-[M−H]−/ M+H]^+^	
Anthocyanins [56]	Plasma; SPE; Malvidin-3-*O*-glucoside	Poroshell 120 C18 (2.1 mm × 150 mm, 2.7 μm); A: Water (1% FA), B: ACN.		
Flavonoids, phenolic acids, stilbenes, lignans, and tyrosol derivatives [106,118]	Urine; SPE; Taxifolin	Luna Omega Polar C18 (100 × 2.1 mm, 1.6 μm); A: 10 mM ammonium formate (0.1% FA), B: ACN.	QTRAP; MRM; ESI- [M−H]^−^	10 μg L^−1^
	Plasma; PP and SPE; Ferulic acid-1,2,3-13C3, L-phenylalanine-15N	Luna Omega Polar C18 (100 × 2.1 mm, 1.6 μm); A: Water, B: ACN, both with 0.5% FA.	QTRAP; MRM; ESI-[M+H]^+^	0.5–5 μmol L^−1^
Anthocyanins [105]	Urine; SPE; Phloridzin, scopoletin, taxifolin, and 7,8-dihydroxycourmarin	Kinetex PFP (4.6 × 100 mm, 2.6 μm); A: Water, B: ACN, both with 0.1% FA.	QTRAP; MRM; ESI-[M−H]^−^	

AA: acetic acid; FA: formic acid; TFA: trifluoroacetic acid; SRM: selective reaction monitoring; SIM: selected ion monitoring, CRM: consecutive reaction monitoring; TIS: turbo ion spray; UD: urine dilution; UU: unprocessed urine; HESI: heated-electrospray ionization source. * d^13^C_2_-caffeic acid, d_3_-dihydroisoferulic acid, and d_3_-dihydroisoferulic-3′-*O*-glucuronide.

### 3.3. GC-MS

GC is generally recognized to be more reproducible, cheaper, and producing higher resolution than LC [112,121]. However, in PC analysis, its limitations have tipped the scales in favor of LC. Since the development of LC, there has been little evolution in GC methodologies, which in recent publications are clearly surpassed by LC [121]. The main disadvantage of GC is that the analytes need to be volatile (or transformed into volatile or semi-volatile compounds) and thermally stable, so their boiling temperature should be lower than 350–400 °C [122]. Secondly, it is necessary to add a derivatization step prior to the injection to generate phenolic derivatives that can be analyzed by GC. This procedure is performed with methylating or silylating reagents, most commonly *N,O*-bis(trimethylsilyl)trifluoroacetamide (BSTFA), trimethylchlorosilane (TMCS), *N*-methyl-*N*-(trimethylsilyl) (MSTFA), 1,1,1,3,3,3-hexamethyldisilazane (HMDS), *N*-methyl-*N*-(tert-butyldimethylsilyl)trifluoroacetamide (MTBSTFA), and tetramethylammonium hydroxide (TMAH) [112].

The combination of GC with MS provides a sensitive technique for the identification and quantification of PCM [121]. Flavonoids possess physicochemical properties that make them especially suitable for GC-MS analysis [123]. Isoflavones and flavonoids and their metabolites were studied in urine samples previously derivatized with MSTFA [124]. Another study identified phase I metabolites of isoflavones after performing the derivatization with pyridine/HMDS/TMCS, 9:3:1 [125].

In 2018, Ordóñez et al. compared the use of GC-MS and HPLC-MS for the analysis of microbial-derived phenolic acids in urine. Interestingly, they observed that GC-MS is not suitable for the analysis of phase-II glucuronide and sulfate metabolites, which are insufficiently volatile. However, other microbial urinary phenolic metabolites, such as caffeic acid, ferulic acid, or isoferulic acid, were detected after using MSTFA as a derivatization agent [126]. Most studies employing GC-MS to analyze PCM have focused on colonic metabolites after the consumption of polyphenol-rich foods. It was used by Draijer et al. to obtain a fingerprint of phenolic acids in urine, employing BSTFA with 10% TMCS to derivatize the metabolites [127]. Other studies used pyridine and MSTFA (1:4, *v/v*) or BSTFA/TMCS (90:10 *v/v*) to achieve the separation and identification of phenolic acids and aromatic acids produced by the gut microbiota [45,63]. The main metabolites of chlorogenic acid, caffeic, and ferulic acid were analyzed in plasma samples after coffee intake. The derivatization of the analytes was carried out with MTBSTF and 1% tert-butyldimethylsilyl chloride, and the products were redissolved in hexane prior to injection [110].

GC-MS/MS was used to characterize the urinary metabolites of olive oil PC in samples derivatized with MSTFA/1-(trimethylsilyl)imidazole (TMSI) 1000/2 (*v/v*), resulting in the identification and quantification of free and conjugated forms of hydroxytyrosol, tyrosol, and elenolic acid [22]. In another study on olive oil, metabolites of hydroxytyrosol, homovanillic alcohol, and homovanillic acid were identified and quantified after their transformation into trymethylsilyl ether derivates [28].

The application of GC to determine PCM is not limited to its coupling with MS. GC-QqQ/MS was applied by Carry et al. to analyze microbial phenolic acid metabolites of grape epicatechin and catechin [128]. Furthermore, Kay et al., employed GC with a flame ionization detector to identify the glycosylating structures of cyanidin, and the derivatization of sugars was performed with TMSI + pyridine [54].

### 3.4. NMR

NMR spectroscopy is a potent tool for the qualitative and quantitative analysis of complex mixtures of small molecules and has been used to analyze PC in food [129,130,131]. NMR-based metabolomics offers a powerful approach to metabolite fingerprinting and profiling [132]. As well as being non-destructive, quantitative, and highly reproducible, this technique requires minimal sample preparation, allows the identification of new compounds, and is information-rich for the characterization of molecular structures, particularly in complex mixture analyses [133]. However, a limiting factor is the complexity of interpreting the NMR spectra of biological samples, due to signal overlap and crowding, so multivariate statistical analysis is essential [129,130,134]. A study in 2005 profiled the flavonol metabolites of black tea in urine samples using ^1^H NMR spectroscopy followed by pattern recognition techniques, which converted the NMR spectra into a set of spectroscopic integrals used as descriptors for principal component analysis [40].

An ^1^H NMR metabolomic approach was also used to profile the metabolomic changes in plasma arising from the consumption of cranberry procyanidins [59]. Similarly, urinary metabolome changes in female Sprague–Dawley rats were examined after the administration of partially purified cranberry or apple procyanidins [135], and recently, an in-depth analysis of the effects of resveratrol on the urinary and fecal metabolome of female Wistar rats [136] was performed. 2D NMR spectra (heteronuclear single-quantum correlation (HSQC) or heteronuclear multiple-quantum correlation (HMQC) spectroscopy) were used in some examples to assist the metabolite identification and confirm the metabolite assignments.

The complexity of the urine matrix, together with the low concentration of the conjugates, which have many possible isomeric forms, requires the occasional use of an SPE sample preparation procedure to both purify and concentrate the PC metabolites before NMR analysis. In this context, Jacobs et al. developed a method in which three complementary metabolite sub-profiles were generated with different compound classes by SPE fractionation [137]. Separation of phenolic from polar metabolites improved the identification by reducing signal overlap in the ^1^H NMR spectra and increasing phenolic concentrations. This SPE-NMR sub-profiling method was tested on urine samples collected from a crossover human nutritional intervention trial in which healthy volunteers consumed a mixture of wine and grape polyphenol extracts or a placebo.

In another study, van der Hooft reported a procedure for the identification and quantification of PC metabolites in urine after intake of tea using a methodology in which MS-based SPE trapping was coupled to ^1^H NMR spectroscopy [138]. A urine clean-up and preconcentration by means of SPE has also been recently employed by de Roo for the spectral assignment of conjugated valerolactone metabolites of catechin-based polyphenols isolated from the urine of black tea consumers [139]. In this study, an SPE-preparative liquid chromatography (prepLC)–MS–LC–MS-NMR workflow allowed the full spectral ^1^H and ^13^C NMR assignments of five conjugated valerolactones, applying 1D and 2D homo- and hetero-nuclear NMR experiments.

Moazzami et al. developed a quantitative NMR analysis to ascertain the pattern of sesamin urinary metabolites in humans. In this study, after collection by HPLC, the major catechol metabolites were characterized by ^1^H-NMR, homonuclear correlation spectroscopy (COSY), total correlation spectroscopy (TOCSY), heteronuclear multiple-bond correlation (HMBC), HSQC, and two-dimensional nuclear Overhauser effect (NOESY) experiments, and the excretion post-consumption of a specific dose of sesame oil was quantified from urine extracts by ^1^H-NMR spectra [140].

While MS/MS and MS^n^ fragmentation can be very helpful in metabolite characterization and identification [133], it is not useful to establish a substitution position, unless the reference of the compounds are available [18]. Therefore, to elucidate their complete structure, additional information from NMR spectra is required. A noteworthy application was reported by Natsume in 2003 [141], where the elucidation of the chemical structure of several (-)-epicatechin metabolites (methylated and glucuronidated forms) in human and rat urine was performed by ^1^H NMR, ^13^C NMR, and HMBC (and LC-MS) analyses. In another study, Miksits et al. located the sulfate at position 3 and identified the full structure of the main resveratrol monosulfated metabolite by NMR experiments (1D ^1^H, 2D double-quantum filtered-COSY, HSQC, and HMBC) [142]. The elucidation of the truly active structures of (+)-catechin and (-)-epicatechin in biological rat fluids was established as (+)-catechin 5-*O*-β-glucuronide and (-)-epicatechin 5-*O*-β-glucuronide by both MS analysis and the full assignment of ^1^H and ^13^C NMR, which was achieved by 2D experiments (COSY, HSQC, and HMBC). In this study, the conjugation position of the glucuronide was established by HMBC spectra and was additionally corroborated by NOESY analysis [143].

## 4. Conclusions

The characterization of PCM in plasma and urine is a strategy to understand the biological effects of PC present in plant-derived feeds, foods, beverages, herbal medicines, and dietary supplements. Due to their beneficial impacts on health, considerable research has been focused on the identification and quantification of PCM in plasma and urine of human and rats after the consumption of dietary PC. During the last two decades, considerable progress has been achieved in sample preparation methods (PCM extraction, cleaning/purification, preconcentration, and derivation), allowing the analysis of PC and PCM with differentiated physicochemical properties. However, problematic issues in sample clean-up include a lack of commercial standards for PCM and internal standards, metabolite degradation due to tedious multi-step sample preparation workflows, instability of specific compounds, and low analyte concentration.

PCM analysis has benefited from recent advances in high-throughput identification by hyphenated chromatographic techniques. The presence of PCM with highly variable physicochemical traits (hydrophilicity, lipophilicity, and protolytic properties), their small structural differences, and low levels of abundance remain a challenge for their identification and quantitation by existing analytical platforms. Currently, there is lack of clear guidelines or strategies for the identification and mainly for the targeted quantification of PCM without standards. Reversed-phase LC is actually the best approach, but the column specificity (particle size or the use of core shell particles), the mobile phase, and additives included in the mobile phases, and the performance of interface with detection systems, especially with the MS, are all important to ensure and sustain data quality, mainly for isomeric or isobaric compounds that are challenging and require both chromatographic separation and mass spectrometry detection.

## Figures and Tables

**Table 1:** Food source of phenolic compounds and the predominant phenolic compound metabolites identified in plasma and urine samples.

Food or Supplement	Compounds	Metabolites	Sample	Ref
VOO/EVOO	Phenolic acids and tyrosols	PI	Plasma	[21]
	Oleuropein		Urine	[22]
	Hydroxytyrosol	PII		[23,24]
	Tyrosols			[25]
	Phenolic acids and tyrosols		Plasma	[14]
	Hydroxytyrosol			[26]
	Flavones, lignans, and tyrosols	PI/PII	Urine	[27]
	Tyrosols			[28]
	Secoiridoids		Plasma/urine	[29]
	Hydroxytyrosol and derivatives	PII/MM		[30,31]
Coffee	Chlorogenic acids	PII/MM	Plasma/urine	[32,33]
	Phenolic acids			[34]
		PI		[35]
		PII	Plasma	[36]
		MM		[37]
Green tea	Flavan-3-ols	PII	Urine	[38]
		PII/MM		[39]
Black tea	Hydroxybenzoic acids			[40]
	Flavonoids and phenolic acids		Plasma/urine	[41]
Yerba mate	Flavonols and phenolic acids			[42]
Orange juice	Flavonoids and phenolic acids	PII	Plasma/urine	[43]
	Flavanones and flavonols		Urine	[44]
	Flavanoids	PII/MM		[45]
	Flavanones			[46]
			Plasma/urine	[47]
Red wine	Flavonoids, phenolic acids, lignans, stilbenes	PII/MM	Urine	[48]
	Stilbenes			[49]
	Proanthocyanidin		Plasma/urine	[50]
Bilberry	Anthocyanins	PII	Plasma/urine	[51]
Black raspberry	Anthocyanins	PII	Urine	[52]
Red raspberry	Flavan-3-ols and phenolic acids	PII/MM	Plasma/urine	[53]
Chokeberry extract	Anthocyanins	PI/PII	Urine	[54]
Strawberry drink	Anthocyanins	PII	Plasma	[55]
	Anthocyanins and phenolic acids	PII/MM		[56]
Cranberry juice	Flavonols, flavan-3-ols, and phenolic acids	PII/MM	Plasma/urine	[57]
	Flavan-3-ols			[58]
Cranberry extract	Procyanidins	PII/MM	Plasma	[59]
Cranberry-syrup	Flavonoids and phenolic acids	PI/PII	Urine	[60]
Grape pomace drink	Flavan-3-ols and phenolic acids	PII/MM	Plasma/urine	[61]
Concord grape juice	Flavonoids and hydroxycinnamic acids	PII	Plasma/urine	[62]
	Phenolic acids	MM	Urine	[63]
Grape extract	Flavonoids, phenolic acids, and stilbenes	PII/MM	Urine	[64]
PRJD	Flavonoids and phenolic acids	PII/MM	Plasma/urine	[65]
PRBF	Flavonoids and phenolic acids	PII	Urine	[66]
Apple	Flavonoids and phenolic acids	PII/MM	Plasma	[67]
	Flavan-3-ol		Urine	[68]
AAPO	Quercetin	PII	Plasma	[69]
Cocoa	Flavan-3-ols and phenolic acids	MM	Urine	[70]
	Flavonoids and procyanidin	PII/MM	Plasma/urine	[71]
	Flavan-3-ols			[72]
Cocoa cream	Flavan-3-ols	PII	Plasma	[73]
Cocoa drink				[74]
			Plasma/urine	[75]
	(-)-Epicatechin		Urine	[76]
Dark chocolate/ chocolate	Flavan-3-ols	PII	Plasma/urine	[77]
	Isoflavones		Urine	[78]
	Flavan-3-ols	PII/MM		[79]
Tomato sauce	Flavonoids and phenolic acids	PII	Plasma/urine	[80]
Tomato sauce with oil	Flavanones and hydroxycinnamic acids			[3]
Tomato/tomato sauce	Flavonoids and phenolic acids	PII/MM		[81]
Almonds	Flavonoids	PII/MM	Plasma/urine	[82]
Beans	Flavonoids and phenolic acids	PII/MM	Plasma/urine	[83]
Black soybean extract	Isoflavones	PII	Plasma/urine	[84]
Standard solutions	Catechin	PII/MM	Urine	[85]
	Quercetin		Plasma/urine	[86]
	(-)-Epicatechin	PI/PII/MM		[87]
	Daidzein			[88]
	Gallic acid	PII		[89]
	Trans-resveratrol		Urine	[90]
	Caffeic, ferulic, and isoferulic acid	PI/PII	Plasma/urine	[91]
	Hesperetin and hesperidin			[92]
	Oleocanthal		Plasma	[93,94]
	Oleacein			[95]
Capsule	(-)-Epicatechin and procyanidins	PII/MM	Plasma/urine	[96]
	Luteolin	PII	Plasma	[97]
Pill	Trans-resveratrol	PII/MM	Plasma	[98]

PI: Phase I metabolites; PII: Phase II metabolites; MM: microbial metabolites; VOO: Virgin olive oil; EVOO: Extra virgin olive oil. PRJD: Polyphenol-rich juice drink, PRBF: Puree of (poly)phenol-rich berry fruits; AAPO: Applesauce with apple peel and onion.

**Table 2:** Methods for untargeted analysis of PCM in urine and plasma samples.

Phenolic Compounds	Sample; Sample Clean-Up; Internal Standard	Chromatographic Conditions	MS Conditions
*Phase-I and Phase-II Metabolites*			
Quercetin [86]	Urine and plasma; PP	Acquity BEH C_18_ (2.1 × 50 mm, 1.7 μm); A: Water (0.1% FA). B: ACN.	QTOF; ESI-[M−H]^−^
Secoiridoids [29]	Urine and plasma; UU and PP	Hypersil gold C_18_ (2.1 × 100 mm, 1.9 μm); A: Water (0.01% AA), B: ACN.	Q-Exactive; ESI-[M−H]^−^
Epicatechin [87]	Urine and plasma; SPE	Acquity BEH C_18_ (2.1 × 100 mm, 1.7 μm); A: Water (0.1% FA), A: ACN:MeOH (3:1 *v/v*).	LTQ-Orbitrap; ESI-[M−H]^−^
Daidzein [88], hesperetin, and hesperidin [92]		Acquity BEH C_18_ (2.1 × 100 mm, 1.7 μm); A: Water (0.1% FA). B: ACN.	LTQ-Orbitrap; ESI-[M−H]^−^/[M+H]^+^
Oleocanthal [93]	Plasma; SPE	Acquity BEH C_18_ (2.1 × 50 mm, 1.7 μm); A: Water, B: MeOH, both with 0.1% FA.	LTQ-Orbitrap; ESI-[M−H]^−^
Phenolic acids and flavonoids [60]	Urine; SPE	Zorbax Eclipse C_18_ (4.6 × 150 mm, 1.8 μm); A: 1% FA in water/ACN (90:10, *v/v*), B: ACN	QTOF; ESI-[M−H]^−^
		Zorbax Eclipse C_18_ (4.6 × 150 mm, 1.8 μm); A: Water (10% FA), B: ACN.	QTOF; ESI-[M+H]^+^
*Phase-II Metabolites*			
Procyanidins [59]	Plasma; PP; mixture solution **	ACE Excel 2 C_18_-PFP (2.1 × 100 mm, 2 μm); A: Water (0.1% FA). B: ACN.	Q-Exactive; HESI-[M−H]^−^
Resveratrol [98]	Plasma; PP; mixture solution *	Nucleodur C_18_ Isis (2 × 150 mm,1.8 μm); A: Water, B: MeOH, both with 0.1% FA.	
Quercetin [69]	Plasma; PP; Quercetin 4′-*O*-glucoside	Poroshell C_18_ (2.1 × 100 mm, 2.7 mm); A: Water, B: ACN, both with 0.1% FA.	QTOF; ESI-[M−H]^−^/ [M+H]^+^
Flavonoids [44]	Urine; UD; Naringin-d4	Kinetex C_18_ (3.0 × 150 mm, 2.6 μm); A: Water, B: MeOH, both with 0.1% FA.	QTOF; ESI-[M−H]^−^
Phenolic acids and flavonoids [66]	Urine; LLE; Rutin and taxifolin	Synergi Hydro RP18 C_18_ (2 × 150 mm, 4 μm); A: Water, B: ACN (0.1% FA).	LTQ-Orbitrap; ESI-[M−H]^−^
Isoflavones [84]	Urine and plasma; SPE	Kinetex C_18_ (2.1 × 100 mm, 1.7 μm); A: Buffer (0.1% FA). B: ACN.	
*Phase-II and Microbial Metabolites*			
Quercetin [86]	Urine and plasma; PP	Acquity BEH C_18_ (2.1 × 50 mm, 1.7 μm); A: Water (0.1% FA). B: ACN,	QTOF; ESI-[M−H]^−^
Phenolic acids and flavonoids [57,83]	Urine and plasma; µSPE; Taxifolin	Zorbax Eclipse Plus RRHD (2.1 × 50 mm, 1.8 µm); A: Water, B: ACN, both with 0.1% FA.	
Phenolic acids and flavonols [42,72]	Urine and plasma; LLE, PP and UD	Ascentis Express C_18_ (3 × 150 mm, 2.7 μm); A: Water, B: ACN, both with 0.1% FA.	
Phenolic acids [32]			
Flavan-3-ol [50]	Urine and plasma; PP	Kinetex Phenyl-Hexyl (4.6 × 150 mm, 2.6 μm); 0.1% FA methanol in 0.1% aqueous FA	Q-Exactive; HESI-[M−H]^−^
Phenolic acids and flavonoids [41]	Plasma; PP; Baicalin and genistein 7-β-d-*O*-glucuronide	Acquity BEH C_18_ (2.1 × 150 mm, 1.7 μm); ACN (0.1% FA).	LTQ-Orbitrap; ESI-[M−H]^−^
Flavanone [47]	Plasma; PP	Kinetex C_18_ (4.6 × 150 mm, 5 μm); 0.1% FA methanol in 0.1% aqueous FA	Q-Exactive; HESI-[M−H]^−^
Phenolic acids and flavonoids [48]	Urine; UU	Zorbax Eclipse Plus C_8_ (2.1 mm × 100 mm, 1.8 μm); A: Water, B: ACN, both with 0.01% FA.	QTOF; ESI-[M−H]^−^
Procyanidins, phenolic acids, and flavonols [64]	Urine; UD	Luna C_18_ (2.0 × 50 mm, 5 μm); A: Water, B: ACN, both with 0.1% FA	LTQ-Orbitrap; ESI-[M−H]^−^
Flavan-3-ol [79]	Urine; SPE: Hesperetin	Poroshell C_18_ (0.5 × 250 mm, 2.7 µm); A: Water, B: ACN, both with 0.1% FA	QTOF; ESI-[M−H]^−^/[M+H]^+^

AA: acetic acid; ACN: acetonitrile; FA: formic acid; UD: urine dilution; UU: unprocessed urine; SPE: solid-phase extraction; PP: protein precipitation; µSPE: micro-elution solid-phase extraction; * L-tryptophan-D3, L-leucine-D10, creatine-D3, and caffeine-D3; ** trans-resvératrol-13C6, trans-resveratrol-3-*O*-sulfate-D4, trans-resveratrol-3-*O*-β-d-glucuronide-D4, and trans-resveratrol-4′-*O*-β-d-glucuronide-D4.

## Supplementary Materials

The following are available online at https://www.mdpi.com/article/10.3390/antiox10060846/s1, Table S1. Secoiridoids metabolites detected in plasma and/or urine from humans and rats; Table S2. Phenolic alcohol metabolites detected in plasma and/or urine from humans and rats; Table S3. Flavonoids metabolites detected in plasma and/or urine from humans and rats; Table S4. Metabolites of phenolic acids detected in plasma and/or urine from humans and rats; Table S5. Metabolites of enterolignans, stilbenes, and ellagitannins detected in plasma and/or urine from humans and rats; Table S6. Metabolites of other phenolic compounds detected in plasma and/or urine from humans and rats.

## Abbreviations

Atmospheric Pressure Chemical Ionization (APCI); Capillary Electrophoresis (CE); Collision-Induced Dissociation (CID); Electrospray Ionization (ESI); Gas Chromatography (GC); Gas Chromatography-Mass Spectrometry (GC-MS); Heteronuclear Single-Quantum Correlation Spectroscopy (HSQC); Heteronuclear Multiple-Bond Correlation Spectroscopy (HMBC); Heteronuclear Multiple-Quantum Correlation Spectroscopy (HMQC); Homonuclear Correlation Spectroscopy (COSY); Liquid Chromatography (LC); Liquid Chromatography-Electrospray Ionization-Mass Spectrometry (LC-ESI-MS); Liquid-liquid Extraction (LLE); Mass Spectrometry (MS); Matrix-Assisted Laser Desorption Ionization (MALDI); Micro-elution Solid-Phase Extraction (µSPE); Nuclear Magnetic Resonance (NMR); N,O-bis(trimethylsilyl)trifluoroacetamide (BSTFA); N-trimethylsilyl-N-methyl trifluoroacetamide (MSTFA); N-methyl-N-(tert-butyldimethylsilyl) trifluoroacetamide and tetramethylammonium hydroxide (TMAH): Phenolic Compounds (PC); Phenolic Compounds Metabolites (PCM); Protein Precipitation (PP); Solid-Phase Extraction (SPE); Solid-Liquid Extraction (SLE); Surface-Induced Dissociation (SID); Total Correlation Spectroscopy (TOCSY); Trimethylchlorosilane (TMCS); Two-dimensional Nuclear Overhauser Effect (NOESY); Ultra-High-Performance Liquid Chromatography-Mass Spectrometry (UHPLC-MS).
