# Supplementary material for: Metabolomics Technologies for the Identification and Quantification of Dietary Phenolic Compound Metabolites: An Overview
Source: Antioxidants (Basel). 2021 May 25;10(6):846. doi: 10.3390/antiox10060846 (PMC8229076; doi:10.3390/antiox10060846)
Supplement: Supplementary file 1 [file antioxidants-10-00846-s001.zip › antioxidants-1202983-supplementary.pdf]

Review

Metabolomics Technologies for the Identification and Quantification of Dietary Phenolic Compound Metabolites: An Overview

Anallely López-Yerena [1,†], Inés Domínguez-López [1,†], Anna Vallverdú-Queralt [1,2], Maria Pérez [1,3], Olga Jáuregui [4,5], Elvira Escribano-Ferrer [2,6,7,*] and Rosa M. Lamuela-Raventós [1,2,*]

[1] Department of Nutrition, Food Science and Gastronomy XaRTA, Institute of Nutrition and Food Safety (INSA-UB), Faculty of Pharmacy and Food Sciences, University of Barcelona, 08028 Barcelona, Spain; naye.yerena@gmail.com (A.L.-Y.); idominguez@ub.edu (I.D.-L.); avallverdu@ub.edu (A.V.-Q.); mariaperez@ub.edu (M.P.)

[2] CIBER Physiopathology of Obesity and Nutrition (CIBEROBN), Institute of Health Carlos III, 28029 Madrid, Spain

[3] Laboratory of Organic Chemistry, Faculty of Pharmacy and Food Sciences, University of Barcelona, 08028 Barcelona, Spain

[4] Scientific and Technological Center of University of Barcelona (CCiTUB), 08028 Barcelona, Spain; ojauregui@ccit.ub.edu

[5] CIBER Fragilidad y Envejecimiento Saludable (CIBERfes), Instituto de Salud Carlos III, 28029, Madrid, Spain

[6] Biopharmaceutics and Pharmacokinetics Unit, Department of Pharmacy and Pharmaceutical Technology and Physical Chemistry, Institute of Nanoscience and Nanotechnology (IN2UB), Faculty of Pharmacy and Food Sciences, University of Barcelona, 08028 Barcelona, Spain

[7] Pharmaceutical Nanotechnology Group I+D+I Associated Unit to CSIC, University of Barcelona, 08028 Barcelona, Spain

† Equally Contributing Authors

* Correspondence: eescribano@ub.edu (E.E.-F.); lamuela@ub.edu (R.M.L.-R.); Tel.: +34-646421580 (R.M.L.-R.)

Table S1. Secoiridoids metabolites detected in plasma and/or urine from humans and rats.

Food or supplement	Compounds	Metabolites	Parent mass	Fragments	Ref
EVOO	Oleuropein and/or oleacein	Hydroxytyrosol	153	123	[1,2]
		Dihydroxyphenyl acetic acid	167	123	[1,3]
		Hydroxytyrosol-glucuronide	329	153	[1]
		Hydroxytyrosol-sulfate	233	153	[1,4]
		Hydroxytyrosol acetate sulfate	275	195	[4]
		Homovanillic acid	181	137	[1,3]
		Homovanillic acid-glucuronide	357	181	[1]
		Homovanillic alcohol	167	152	[1,3]
		Homovanillic alcohol-sulfate	247	167	[1]
		Elenolic acid	241	139	[1,3]
		Elenolic acid-sulfate	321	241	
		Elenolic acid-glucuronide	417	241	[1]
		Methyloleuropein aglycone-sulfate	471	391	
		Oleuropein aglycone-glucuronide	553	377	
		Oleacein	319	195	[5]
		Oleacein-sulfate	399	319	
		Oleacein-glucuronide	495	319/153	[5,6]
	Oleocanthal	Oleocanthal-hydrate	319	153	[7]
		Oleocanthal-H_2O	321	201	
		OLC-hydrate-glucuronide	481	217	
		OLC-H_2O-glucuronide	497	321	

EVOO: Extra virgin olive oil.

Table S2. Phenolic alcohol metabolites detected in plasma and/or urine from humans and rats.

Food or supplement	Compounds	Metabolites	Parent mass	Fragments	Ref
EVOO	Hydroxytyrosol	Hydroxytyrosol-glucuronide	329	153	[1,2,5,8–12]
		Hydroxytyrosol-sulfate	233	153/123	[1,2,4–6,8,10–12]
		Hydroxytyrosol acetate-sulfate	275	195	[4,5,10,12]
		Hydroxytyrosol acetate-glucuronide	371	195	[5]
		Homovanillic alcohol	167	151/91	[5,13]
		Homovanillic alcohol-glucuronide	343	167	[5,9,10,12]
		Homovanillic alcohol-sulfate	247	167	[5,10,12,13]
		Homovanillic acid	181	137	[1,2,5,6]
		Homovanillic acid-sulfate	261	181	[2,4–6,10–12]
		Homovanillic acid-glucuronide	357	181	[5,12]
		Hydroxytyrosol acetate	195	91/123	[5,13]
		Dihydroxyphenylacetic acid	169	123/110	[13]
		Tyrosol	139*	121/91	
	Tyrosol	Tyrosol-glucuronide	313	137	[2,9]
		Tyrosol-sulfate	217	137	[2,6,11]

EVOO: Extra virgin olive oil. *In positive mode.

Table S3. Flavonoids metabolites detected in plasma and/or urine from humans and rats.

Food or supplement	Compounds	Metabolites	Parent mass	Fragments	Ref
		Flavan-3-ols			
Cocoa, cocoa drink, wine, grape extract or epicatechin standard	(Epi)catechin	(Epi)catechin	289	245/205/179/137/151	[14–19]
Black tea, cocoa, cocoa cream and drink, chocolate, dark chocolate, grape (extract and pomace drink), almond, (epi)catechin standard, polyphenol-rich juice, puree of (poly)phenol-rich berry fruits.		(Epi)catechin-glucuronide	465	289/245/205/179/203/289/245	[15–29]
		(Epi)catechin-sulfate	369	289/303/217/137/245	[15–19,22–26,28–30]
Cocoa drink, grape (pomace drink and extract, polyphenol-rich juice		(Epi)catechin-glucuronide-sulfate	545	465/369/289	[19,27,28,30]
(Epic)catechin standard		Methyl-(Epi)catechin	305	179/147/139/137	[18,20]
Cocoa cream, catechin standard, Cocoa, cocoa drink, dark chocolate, chocolate, almond, grape extract, epicatechin standard, cranberry extract		Methyl-(Epi)catechin-glucuronide	479	179/139//137/303/289/175/461	[15–21,24–26,31]
Cocoa cream, catechin standard Black tea, cocoa, cocoa cream and drink, chocolate, dark chocolate, grape (extract and pomace drink), almond, (epi)catechin standard, polyphenol-rich juice, puree of (poly)phenol-rich berry fruits.		Methyl-(Epi)catechin-sulfate	383	289/303/245/217/137	[15,16,18–20,22,24–27,30]
Cocoa drink, chocolate		Methyl-(Epi)catechin-glucuronide-sulfate	559	289	[24,30]
Chocolate		Methyl-(Epi)catechin-diglucuronide-sulfate	639	383	[24]
Cranberry syrup		Catechin-hydrate	287	/	[32]
		Catechin-hydrate-glucuronide	463	/	
Chocolate, epicatechin standard		(Epi)catechin-glucoside	451	289/313/161	[18,24]
Epicatechin standard		(Epi)catechin-sulfate-glucoside	531	369/289/451/313	[18]
Grape extract		(Epi)gallocatechin	305	225	[19]
Polyphenol-rich juice		(Epi)gallocatechin-glucuronide	481	305	[28]
Black tea		Dimethyl-(Epi)gallocatechin-sulfate	413	/	[30]
Grape extract, polyphenol-rich juice		Methyl-epigallocatechin-glucuronide	495	319	[19,28]
Almond, green tea		Hydroxyphenyl-γ-valerolactone	191	147	[26,33]
Green tea, chocolate		Hydroxyphenyl-γ-valerolactone-glucuronide	367	207/197/191	[24,33]
Green tea, epicatechin standard, chocolate, almond		Hydroxyphenyl-γ-valerolactone-sulfate	271	191	[18,24,26,33]
Green tea, grape extract, epicatechin standard, chocolate, almond, concord grape juice		Dihydroxyphenyl-γ-valerolactone	207	163/161/147/ 135/122/109	[18,19,24,26,33,34]
Green tea, chocolate, almond, epicatechin standard		Dihydroxyphenyl-γ-valerolactone-glucuronide	383	207/175/163/135/122/113	[24,26]
Green tea, epicatechin standard		Dihydroxyphenyl-γ-valerolactone-sulfate	287	207	[18,33]
Green tea		Dihydroxyphenyl-γ-valerolactone-di-sulfate	367	287	[33]
		Trihydroxyphenyl-γ-valerolactone	223	179	
Catechin standard		Methoxy-hydroxyphenyl-γ-valerolactone	223	205/164/163	[21]

		Methoxy-hydroxyphenyl-γ-valerolactone-glucuronide	399	381/223/205	
		Methoxy-hydroxyphenyl-γ-valerolactone-sullfate	303	285/223/163	
Epicatechin standard		Dihydroxyphenyl-γ-valeric acid-sulfate	289	209/147/101	[18]
		Trihydroxyphenyl-γ-valeric acid-sulfate	305	225/207/123	
		Methyl-epicatechin-glucoside-sulfate	545	383/303/245/327/501/217	
Green tea, chocolate, almond, epicatechin standard		Methoxyl-hydroxyphenyl-γ-valerolactone-glucuronide	397	175/221/206/177/162/123	[18,24,26,33]
Almond, epicatechin standard		Methoxyl-hydroxyphenyl-γ-valerolactone-sulfate	301	221	[18,26]
Almond		Hydroxyphenylpropionic acid	165	121	[26]
Epicatechin standard		Hydroxyphenylpropionic acid-glucuronide	341	175/165/121/113	[18]
		Hydroxyphenylpropionic acid-sulfate	245	165/121	
Almond		Dihydroxyphenylpropionic acid	181	137	[26]
Epicatechin standard		Methyl-dihydroxyphenylpropionic acid-sulfate	275	195	[18]
		Dihydroxyphenylacetic acid-sulfate	261	181/137/217	
		Hydroxyphenylacetic acid-sulfate	231	151/217	
		Benzonic acid-sulfate	217	137	
		Tetrahydroxyphenylpropanol-glucuronide	451	275/217	
		Tetrahydroxyphenylpropanol-sulfate	355	275	
		Pentahydroxyphenylpropanol-sulfate	371	291/247/353/245	
		Methyl-pentahydroxyphenylpropanol-sulfate	385	305	
		Methyl-pentahydroxyphenylpropanol-glucuronide	481	305	
		Methyl-dihydroxyphenylacrylic acid-sulfate	273	193/229	
Almond	Isorhamnetin	Isorhamnetin-glucuronide	491	315	[26]
		Isorhamnetin-sulfate	395	315	
Green and black tea	Epigallocatechin/ epicatechingallate/ epigallocatechingallat e	Methyl-epicatechin-sulfate	383	/	[30,35]
Green tea, grape extract		Epigallocatechin-glucuronide	481	305	[14,15,19]
Black tea		Methyl-epigallocatechin-sulfate	399	/	[30]
		Epicatechingallate-sulfate	521	/	
		Epicatechingallate-glucuronide-sulfate	697	/	
		Methyl-epicatechingallate-sulfate	535	/	
		Methyl-epicatechingallate-glucuronide-sulfate	711	/	
		Epigallocatechingallate-sulfate	537	/	
		Methyl-epigallocatechingallate-sulfate	551	/	[36]
Black tea, yerba mate, orange juice, cranberry juice, strawberry, beans	Kaempferol	Kaempferol-glucuronide	461	363/285/257/229/175/113/85	[30,35,37–41]
Wine	Phenylvalerolactones	Phenyl-γ-valerolactone-glucuronide	367	191	[42]
		Phenyl-γ-valerolactone-sulfate	271	191	
Cocoa	Proanthocyanidin	Dihydroxyphenyl-γ-valerolactone	207	163	[15]
		Methoxy-hydroxyphenyl-γ-valerolactone	221	163	
	Hydroxyphenyl-valerolactones	Dihydroxyphenyl-γ-valerolactone-glucuronide	383	207	
		Dihydroxyphenyl-γ-valerolactone-sulfate	287	207	
		Methoxy-hydroxyphenyl-γ-valerolactone-glucuronide	397	221	
Cocoa, chocolate		Methoxy-hydroxyphenyl-γ-valerolactone-sulfate	301	221/206/162/161/147	[15,24]
Chocolate		Methoxy-hydroxyphenyl-γ-valerolactone-sulfate	317	222/179/163/137/125/113	[24]

		Dihydroxyphenyl-γ-valerolactone-sulfate	303	165/136/124/123/121	
Cocoa, wine		Procyanidin B1	577	425,289	[14,42]
Wine		Procyanidin B2	577	425, 289	
Cranberry syrup	Myricetin	Myricetin	317	/	[32]
		Methyl-myricetin	331	317	
		Methyl-myricetin-glucuronide	507	317	
Tomato sauce, cranberry syrup, grape extract, beans	Quercetin	Quercetin	301	151	[19,32,41,43]
Tomato sauce, applesauce enriched with apple peel and onion, quercetin standard		Quercetin-sulfate	381	301/151	[43–45]
Tomato sauce, cranberry (juice and syrup), applesauce enriched with apple peel and onion, quercetin standard		Quercetin-glucuronide	477	301/151	[32,38,43–45]
Applesauce enriched with apple peel and onion, quercetin standard		Quercetin-diglucuronide	653	477/301	[44,45]
		Quercetin-glucuronide-sulfate	557	477/380/301	
Quercetin standard, cranberry syrup		Methyl-quercetin	315	300/151	[32,44]
Yerba mate, applesauce enriched with apple peel and onion, quercetin standard, cranberry syrup		Methyl-quercetin-glucuronide	491	315/300	[32,44–46]
Applesauce enriched with apple peel and onion or quercetin standard		Methyl-quercetin-diglucuronide	667	491/315/300	[44,45]
Quercetin standard		Methyl-quercetin-sulfate	395	380/301	[44]
		Methyl-quercetin-glucuronide-sulfate	571	491/315/301	
		Dimethyl-quercetin	329	315/301	
		Dimethyl-quercetin-glucuronide	505	491/315/301	
Cranberry syrup		Methoxy-quercetin-galactoside	477	/	[32]
Grape extract		Dihydroquercetin	303	223	[19]
Applesauce enriched with apple peel and onion		Quercetin-glutathione	651	/	[45]
Flavones					
EVOO	Apigenin	Apigenin-glucuronide	445	269	[6,11]
Luteolin standard	Luteolin	Luteolin-glucuronide	461	285	[47]
Isoflavones					
Daidzein standard	Daidzein	Equol	241	212/135/121	[48]
Daidzein standard, chocolate		Equol-glucuronide	417	175/241/113	[48,49]
Chocolate	Equol	Equol-sulfate	321	241	[49]
Black soybean or daidzein standard	Daidzein	Genistein	269	225/181/201	[48,50]
Chocolate, black soybean	Genistein	Genistein-glucuronide	445	269/175	[49,50]
		Genistein-sulfate	349	269/303/225	
Black soybean		Hydroxygenistein-glucuronide	461	286/175	[50]
	Daidzein	Daidzein	253	225/209/197	
Chocolate, black soybean, daidzein standard		Daidzein-glucuronide	429	253/175	[48–50]
		Daidzein-sulfate	333	253	
Black soybean		Daidzein-disulfate	413	333/253	[50]
		Daidzein-glucuronide-sulfate	509	333/253	
Daidzein standard		Daidzein-hydrate	255	135/254/149	[48]

		Daidzein-dihydrate	257	137/161/147	
Black soybean		Hydroge-daidzein-glucuronide	431	255/175	[50]
		Hydroge-daidzein-sulfate	335	255/253/207	
Daidzein standard		Methyl-daidzein	267	252	[48]
		Daidzein-glucoside	415	253/252/295	
		Daidzin-glucoside	579	2755/481	
Black soybean, daidzein standard		Daidzin	415	253/191/155	[48,50]
Black soybean		Daidzin-sulfate	495	415/253	[50]
Daidzein standard		Puerarin	415	295	[48]
		Methyl-genistein	283	268	
		Dimethyl-genistein	285	270/149	
		Glucosygenistin	593	269/431/433/271/178	
		Hydroxygenistein	285	257/229/217	
		Hydroxygenistein-glucosides	447	285/327/255	
		Glycitein	283	268/240	[50]
		Glycitein-glucuronide	459	283/268/240	
		Glycitein-sulfate	363	283/268/240	
		Dihydroxyphenyl-chromane-diol	275	151	[48]
		Hydroxyphenyl-chromene-triol.	271	165/151	
		Hydroxyphenyl-chromen-ol	239	147/195/197	
Flavanones					
Tomato sauce, orange juice	Naringenin	Naringenin	271	253/235//151/119/107/93/83	[37,43]
Tomato sauce, almond		Naringenin-glucuronide	447	271	[26,43,51]
Orange juice		Naringenin-diglucuronide	623	447/313/271/1757151/113	[37,52]
		Naringenin-glucoside-glucuronide	609	489/447/429/313/271/175/151/113	[37]
		Naringenin-glucoside-sulfate	513	433/351/313/271/187/177/151/119	
		Naringenin-sulfate	351	271/177/164/119/107/93	
		Methylnaringenin-glucuronide	461	285/270/243/175/164/151/113/85	
Orange juice or hesperetin standard	Hesperetin	Hesperetin	301	286/255/242/215/201/164/151/136	[37,53]
Orange juice, polyphenol-rich juice, hesperetin standard		Hesperetin-glucuronide	477	301/175/151/459	[28,37,52,53]
Orange juice, hesperetin standard		Hesperetin-diglucuronide	653	477/301/286/242/175/113/85	[37,52,53]
		Hesperetin-sulfate	381	301/286/257/242/229/164/151/149	[37,53]
Orange juice		Hesperetin-disulfate	461	381/365/301/286/231/113	[37]
Orange juice, polyphenol-rich juice		Hesperetin-glucuronide-sulfate	557	477/381/301/286/254/242/229/113	[28,37,52]
Hesperetin standard		Hesperetin-glucoside	463	175/445/151	[53]
Orange juice		Hesperetin-sulfate-glucoside	543	463/423/381/373/301/286/257/242	[37]
		Isosakuranetin-glucuronide	461	285	[52]
		Dihesperetin-glucuronide	955	477/301/175/113/85	[37]
Hesperidin standard	Hesperidin	Hesperidin	609	301/286/257	
		Hesperidin-glucuronide	477	301/459	
		Hesperidin-sulfate	381	229/149/134/151	
		Hesperidin-diglucuronide	653	477/301	

		Hesperidin-glucoside	611	413/197/443	
		Hesperidin-demethoxylate	433	271/256/257//415	
		Hesperidin-demethylate	595	577/559/529/487	
Orange juice	Eriodictyol	Eriodictyol-glucuronide-sulfate	543	463/367/287/254/187/175/151/135	
		Eriodictyol-glucuronide	463	427/287/151/135/113/85	
		Eriodictyol-sulfate	367	287/151/135/107	
		Homoeriodictyol	301	286/242/201/174/151/136/108/107	
		Anthocyanidins			
Chokeberry extract, strawberry drink	Cyanidin	Cyanidin-glucoside	449	287	[36,39,54,55]
		Cyanidin-glucuronide	463	287	[54]
Strawberry drink	Pelargonidin	Pelargonidin-glucuronide	447	/	[36,39,40]
		Pelargonidin-glucoside	433	271/121	
		Pelargonidin-rutinoside	579	433/271	
Bilberry	Peonidin-arabinoside	Delphinidin-glucuronide	479	303	[56]
	Malvidin	Malvidin-arabinoside-glucuronide	639	331	
	Cyanidin	Peonidin-glucoside-glucuronide	639	301	
	Petunidin	Malvidin-glucoside-glucuronide	669	339	
	Peonidin	Petunidin-glucoside-glucuronide	655	317	
Red raspberry	Anthocyanins	Hydroxybenzoic acid	137	93	
		Hydroxybenzoic acid-sulfate	233	153	
Red raspberry, yerba mate		Dihydroxybenzoic acid	153	109	[46,55]
Red raspberry		Ferulic acid-glucuronide	369	351/325/193/175/149/134/113/178	[55]
		Hippuric acid	178	134	
		Hydroxyhippuric acid	194	100/150	
Almond		Phenylacetic acid	135	91	[26]
		Hydroxyphenylacetic acid	151	107	
Red raspberry, almond		Dihydroxyphenylacetic acid	167	123/137	[26,55]
		Methoxy-hydroxyphenylacetic acid	181	137/109/121	
Red raspberry		Dihydrocaffeic acid-sulfate	261	181/109/121	[55]
		Ferulic acid	193	134/178/149	
		Ferulic acid-glucuronide	369	351/325/193/175/149/134/113/178	
		Isoferulic acid-glucuronide	369	193/178/113/134//175	
		Isoferulic acid-sulfate	273	193/178/134/149	
Cranberry extract		Peonidin-glucoside	461	264	[31]
		Dihydrochalcones			
Polyphenol-rich juice, apple	Phloretin	Phloretin-glucuronide	449	273	[28,57]
Polyphenol-rich juice		Phloretin-glucuronide-sulfate	529	353/449/273	[28]

Table S5. Metabolites of phenolic acids detected in plasma and/or urine from humans and rats.

Food or supplement	Compounds	Metabolites	Parent mass	Fragments	Ref
Hydroxybenzoic acids					
Cocoa, EVOO, cranberry juice		Vanillic acid	167	152/123	[6,14,15,38]
Orange juice, grape pomace drink, puree of (poly)phenol-rich berry fruits		Vanillic acid-glucuronide	343	167	[27,29,52]
EVOO, orange juice, cranberry juice, grape pomace drink, puree of (poly)phenol-rich berry fruits, grape extract		Vanillic acid-sulfate	247	203, 167, 123	[6,11,19,27,29,38,41,52]
Cranberry juice		Isovanillic acid	167	/	[38]
Cocoa, grape extract, cranberry juice, almond, beans		Protocatechuic acid	153	109	[14,15,19,26,38,41]
Grape extract		Protocatechuic-glucoside	315	153	[19]
Grape pomace drink	Protocatechuic acid	Protocatechuic acid-glucuronide	329	153	[27]
Grape pomace, puree of (poly)phenol-rich berry fruits		Protocatechuic acid-sulfate	233	153	[27,29]
Concord grape juice		Mandelic acid	151	73	[34]
	Benzoic acid	Benzoic acid	105	77	
Grape pomace drink		Benzoic acid-sulfate	217	137	[27]
Cocoa, coffee, beans, orange juice, red raspberry, strawberry, wine, cranberry (juice, syrup, and extract), almond, grape and concord grape juice		Hydroxybenzoic acid	137	93	[14,15,19,26,27,31,32,32,34, 36,38,41,42,52,58]
Coffee, cranberry juice, cranberry syrup		Dihydroxybenzoic acid	153	109	[32,38,58]
Coffee, concord grape juice		Methoxy-hydroxybenzoic acid	167	123	[34,58]
Tomato sauce, black tea, orange juice, wine, cranberry (juice and syrup), grape (extract, pomace drink, and juice) and beans	Benzoic acid	Hippuric acid	178	134	[19,27,30,32,34,38,41–43,52,59]
Almond, black tea, beans. cocoa, coffee, orange juice, tomato sauce, wine, yerba mate, cranberry (juice and syrup), and grape (extract, pomace drink, and juice).	Hydroxybenzoic acid	Hydroxyhippuric acid	194	100	[14,19,26,27,30,32,34,38,41–43,46,52,58,59]
Wine, cranberry juice, grape pomace drink.		Gallic acid	169	125	[19,27,38]
Puree of (poly)phenol-rich berry fruits.		Gallic acid-glucuronide	345	169	[29]
Grape extract, puree of (poly)phenol-rich berry fruits		Methyl-gallic acid	183	139/95	[19,29]
Black tea, cranberry juice, grape extract, polyphenol-rich juice, puree of (poly)phenol-rich berry fruits	Gallic acid	Methyl-gallic acid-sulfate	263	183	[19,28–30,38]
Grape extract		Dimethyl-gallic acid	197	182/153/137	[19]
Cranberry juice, grape extract		Syringic acid	197	137	[19,38]
Hydroxycinnamic acids					
Cocoa, wine, cranberry juice, almond, coffee, beans, grape extract		Coumaric acid	163	119/91	[14,15,19,26,38,41,60]
Wine, coffee	Coumaric acid	Coumaric acid-glucuronide	339	163/113/119	[42,58,60]
EVOO, coffee		Coumaric acid-sulfate	243	199/163/119	[6,11,58,60,61]
Coffee, yerba mate		Dihydrocoumaric acid	165	121/106/59	[46,58,60,62]
		Dihydrocoumaric acid-glucuronide	341	113/121/59/165	[46,58,60]

		Dihydrocoumaric acid-sulfate	245	165/121/59	[46,58,60,62]
		Dihydrocoumaroylquinic acid	339	165	[46,58]
Wine, cranberry syrup		Coumaroyl-glucose	325	/	[32,63]
Coffee, yerba mate		Coumaroylquinic acid	337	163	[46,58]
Cocoa, coffee, EVOO, tomato sauce, wine, grape extract, cranberry juice, almond, beans, standard solution		Caffeic acid	179	135	[6,14,15,19,26,38,41–43,51,58,60,63,64]
Tomato sauce, coffee, yerba mate, grape extract, EVOO, polyphenol-rich juice, puree of (poly)phenol-rich berry fruits	Caffeic acid	Caffeic acid-sulfate	259	215/179/135	[6,19,28–30,43,58,60–62]
Tomato sauce, wine, cranberry juice, beans, standard solutions		Caffeic acid-glucuronide	355	311/179/135	[38,41–43,51,60,61,64]
Cranberry syrup		Caffeic acid-glucoside	341	179	[32]
		Caffeic acid-glucoside hydrate	339	/	
Standard solution		Hydroxyglucuronyl crylic acid	355	337/311/293/179/175/135/113	[64]
		Glucuronylhydroxy crylic acid	355	337/311/267/179/175/135/113	
Tomato sauce, coffee, yerba mate, grape extract, cranberry juice	Caffeic acid	Dihydrocaffeic acid	181	137/109	[38,43,46,58,60,62]
Tomato sauce, coffee, yerba mate, cranberry juice, grape pomace drink, beans, polyphenol-rich juice	Dihydrocaffeic acid	Dihydrocaffeic acid-sulfate	261	181/113	[27,28,38,41,43,46,58,60,62]
Tomato sauce, coffee, yerba mate, grape extract, cranberry juice		Dihydrocaffeic acid-glucuronide	357	339/313/295/181/175/166/137/113	[19,38,43,46,58,60,61]
Coffee, yerba mate	Caffeic acid	Caffeoylquinic acid	353	191/179	[46,58,60,62]
Coffee		Caffeoylquinic-sulfate	433	191/353/179	[60]
		Caffeoylquinic-lactone	335	161/133	
Coffee, yerba mate		Dihydrocaffeoylquinic acid	355	181	[46,58]
Yerba mate		Caffeoylquinic lactone-sulfate	415	335	[46]
Cocoa, tomato sauce, coffee, wine, cranberry juice, almond, standard solution	Undefined or ferulic acid<br>Ferulic acid	Ferulic acid	193	134/178/149	[14,15,26,38,42,43,51,58,60,64]
Tomato sauce, coffee, yerba mate, orange juice, EVOO, cranberry juice, beans, polyphenol-rich juice, puree of (poly)phenol-rich berry fruits, grape extract		Ferulic acid-sulfate	273	193/149/134/178	[6,19,28,29,38,41–43,46,52,58,60–62]
Tomato sauce, coffee, yerba mate, orange juice, EVOO, cranberry juice, grape pomace drink, puree of (poly)phenol-rich berry fruits		Ferulic acid-glucuronide	369	351/325/193/175/149/134	[6,27,29,38,41–43,46,51,52,58,60–62]
Coffee		Methyl-ferulic acid	207	103/163	[60,62]
		Methyl-dihydroferulic acid	209	150/165	[60]
Yerba mate, cranberry juice, coffee, polyphenol-rich juice		Dihydroferulic acid	197	136	[28,38,43,46,58,60,62]
Yerba mate, grape extract, cranberry (juice and syrup), coffee, beans		Dihydroferulic acid-glucuronide	373	195/175/136	[19,32,38,41,43,46,60,62]

Yerba mate, grape extract, coffee, orange juice, beans, (poly)phenol-rich berry fruits		Dihydroferulic acid-sulfate	277	195	[19,28,38,41,43,46,52,60,62]
Tomato sauce, coffee, yerba mate, cranberry juice, standard solution		Isoferulic acid	193	178/134	[38,43,46,58,60,62,64]
Coffee, yerba mate, orange juice, wine, cranberry juice, puree of (poly)phenol-rich berry fruits		Isoferulic acid-glucuronide	369	193/134	[29,38,42,46,52,58,60,62]
Coffee, yerba mate, polyphenol-rich juice		Dihydroisoferulic acid	195	136	[28,46,58]
Coffee, yerba mate, orange juice, cranberry juice, beans		Dihydroisoferulic acid-glucuronide	371	195/136	[38,41,46,52,58]
Coffee, yerba mate, EVOO, cranberry juice		Dihydroisoferulic acid-sulfate	273	195/136	[11,38,46,58]
Coffee, yerba mate		Feruloylquinic acid	367	191	[46,58,60,62]
Coffee		Feruloylquinic-sulfate	447	191/193	[60]
		Feruloylquinic-glucuronide	543	191/193	
		Feruloylquinic-lactone	349	175/160	
Coffee, yerba mate		Dihydroferuloylquinic acid	369	195	[46,58]
Coffee		Feruloylquinic lactone-glucuronide	525	349	[58]
Coffee, yerba mate, orange juice, grape pomace drink, polyphenol-rich juice		Feruloylglycine	250	191/134	[27,28,46,52,58]
Coffee, yerba mate		Isoferuloylglycine	250	191/134	[46,58]
Cranberry juice, beans		Sinapic acid	223	/	[38,41]
Grape pomace drink		Sinapic acid-sulfate	303	223	[27]
Coffee		Cinnamic acid	147	103	[60]
		Methoxy-cinnamic acid	177	133/117	
	Dimethoxy-cinnamic acid	Dimethoxy-cinnamic acid	207	103	[58]
		Dimethoxy-dihydrocinnamic acid	209	/	
Almond		Methoxy-hydroxycinnamic acid	193	134	[26]
Tomato sauce, cranberry juice.		Chlorogenic acid	353	191	[38,43]
Cranberry syrup		Hydroxychlorogenic acid	369	/	[32]
		Chlorogenic acid + H_2O	371	353	
Coffee	Chlorogenic acid	Caffeoylquinic acid lactone-sulfate	415	335/161/135	[65]
		Feruloylquinic acid	367	193	
		Ferulic acid-sulfate	273	193/178/149/134	
		Feruloylglycine	250	206/191/177/149	
		Dihydroferulic acid-sulfate	277	195/171/151	
		Dihydroferulic acid-glucuronide	371	195/173/113	
		Isoferulic acid-sulfate	273	193/178	
		Isoferulic acid-glucuronide	369	193/175/113	
		Dihydro(iso)ferulic acid-glucuronide	371	195/175/113	
		Caffeic acid-sulfate	259	179/135	
		Dihydrocaffeic acid	181	137/119	
		Dihydrocaffeic acid-glucuronide	357	181/175/137/113	
		Dihydrocaffeic acid-sulfate	261	181/137/119	
Hydroxyphenilacetic acids					

Cocoa, coffee, wine, grape extract, cranberry juice, concord grape juice, wine	Hydroxyphenylacetic acid	Hydroxyphenylacetic acid	151	107	[14,15,19,34,38,38,42,58]
Tomato sauce		Hydroxyphenylacetic acid-sulfate	231	213/187/151/143	[51]
Cocoa, yerba mate, coffee, grape extract, cranberry juice, concord grape juice		Dihydroxyphenylacetic acid	167	123/137	[14,15,19,34,38,46,58]
Orange juice		Dihydroxyphenylacetic acid-sulfate	247	167	[52]
Cocoa, yerba mate, wine, coffee, concord grape juice, wine	Dihydroxyphenylacetic acid	Methoxy-hydroxyphenylacetic acid	181	137	[14,15,34,34,42,46,58]
Black soybean		Hydroxymethoxyl-phenylacetic acid-sulfate	261	181/125	[50]
Concord grape juice		Phenoxyacetic acid	151	165/147/135/73	[34]
Cocoa, cranberry juice		Phenylacetic acid	135	91	[14,15,38]
Grape extract, cranberry juice		Homovanillic acid	181	137	[19,38]
Grape extract, beans, cranberry juice		Homovanillic acid-sulfate	261	181	
Hydroxyphenylpropanoic acids					
Coffee	Phenylpropionic acid	Phenylpropionic acid	149	105	[60]
Wine		Hydroxyphenylpropionic acid- sulfate	245	165	[42]
		Hydroxyphenylpropionic acid-glucuronide	341	165	
Coffee		Methoxy-phenylpropionic acid	179	135	[60]
Wine		Methoxy-hydroxyphenylpropionic acid-sulfate	275	195	[42]
		Methoxy-hydroxyphenylpropionic acid-glucuronide	371	195	
Wine, orange juice		Methoxy-hydroxyphenylpropionic acid	195	136	[42,59]
Tomato sauce, cocoa; yerba mate, wine, coffee, concord grape juice, cranberry juice, standard solution		Hydroxyphenylpropionic acid	165	121	[15,34,42,43,46,51,58]
Tomato sauce, wine, grape pomace drink	Hydroxyphenyl propionic acid	Hydroxyphenylpropionic acid-sulfate	245	227/201/165/121	[27,42]
Wine, orange juice	Dihydroxyphenylpropionic acid	Dihydroxyphenylpropionic aci- glucuronide	357	181	[42,66]
Black tea	Homovanillic acid	Hydroxymethoxyphenylcarboxylic acid-sulfate	261	/	[30]
Cocoa, concord grape juice, cranberry juice, orange juice		Dihydroxyphenylpropionic acid	181	137	[14,15,34,38,59]
Apple, orange juice		Dihydroxyphenylpropionic acid-sulfate	261	181	[57,66]
Standard solution		Dihydroxyphenylmethyoxypropionic acid	211	184/167/123	[64]
		Dihydroxyphenyllactic acid	197	179/153/135/73	
		Trihydroxyphenyllactic acid	213	195/169/151	
		Hydroxymethylphenyl lactic acid	211	193/165/123	
		Hydroxymethyloxyphenyl lactic acid	211	193/148/134	
		Dihydroxyphenyllactic acid methyl ester	211	193/182/165/156	
Hydroxyphenylpentanoic acids					
Cranberry juice, grape pomace drink, apple		Phenyl-γ-valerolactone-sulfate	271	191	[27,67,68]
		Phenyl-γ-valerolactone-glucuronide	367	191	
Grape pomace drink, apple		Phenyl γ-valerolactone-glucuronide-sulfate	463	287/207	[27,68]
Cocoa		Hydroxyphenyl-γ-valerolactone	191	147	[22]
Black tea, cocoa, cranberry extract		Hydroxyphenyl-γ-valerolactone-sulfate	271	191	[22,30,31]

Black tea, cocoa	Hydroxyphenyl-γ-valerolactone	Hydroxyphenyl-γ-valerolactone-glucuronide	367	191	[22,30]
Cocoa, cranberry juice, grape pomace drink		Dihydroxyphenyl-γ-valerolactone	207	163	[22,27,67]
Cranberry juice, apple, grape pomace drink, black tea, cocoa		Dihydroxyphenyl-γ-valerolactone-sulfate	287	207	[22,30,38,67,68]
Cranberry juice, grape pomace drink, apple, black tea		Dihydroxyphenyl-γ-valerolactone-glucuronide	383	207	[27,30,67,68]
Black tea, cranberry juice, cocoa		Dihydroxyphenyl γ-valerolactone-glucuronide-sulfate	463	287	[30,67]
Black tea		Trihydroxy-γ-valerolactone-sulfate	303	/	[30]
		Trihydroxy-γ-valerolactone-glucuronide	399	/	
		Trihydroxy-γ-valerolactone-glucuronide-sulfate	479	/	
Cocoa, black tea, cranberry juice		Methyl-dihydroxyphenyl-γ-valerolactone-sulfate	301	221	[22]
Cocoa, black tea		Methyl-dihydroxyphenyl-γ-valerolactone-glucuronide	397	221	[22,30]
Black tea		Methyl-trihydroxyphenyl-γ-valerolactone-sulfate	317	/	[30]
		Methyl-trihydroxyphenyl-γ-valerolactone-glucuronide	413	/	
Cranberry juice, grape pomace drink, apple		Phenyl-γ-valerolactone-methoxy-sulfate	301	221/206	[27,67,68]
Cranberry juice, apple		Phenyl-γ-valerolactone-methoxy-glucuronide	397	221	[67,68]
Chocolate, cranberry extract	Trihydroxyvaleric acid/ Tetrahydroxyvaleric acid	Dihydroxyphenylvaleric acid	209	193/179/150/137	[24,31]
Cocoa, chocolate		Trihydroxyvaleric acid	225	170/163/147/135	[22,24]
Cranberry extract		Dihydroxyvaleric acid-sulfate	289	/	[31]
Black tea, cocoa, chocolate, apple		Trihydroxyvaleric acid-sulfate	305	225/208/163/148	[22,24,30,68]
		Trihydroxyvaleric acid-glucuronide	401	225/207/163/123	
Black tea		Methyl-dihydroxyphenylvaleric acid-sulfate	303	/	[30]
Black tea, chocolate		Methyl-trihydroxyphenylvaleric acid-sulfate	319	239/163/123/95/79	[24,30]
Black tea		Methyl-tetrahydroxyphenylvaleric acid-glucuronide	431	/	[30]
Apple		Methoxy-hydroxyphenylvaleric acid-sulfate	319	239	[68]

Table S5. Metabolites of enterolignans, stilbenes and ellagitannins detected in plasma and/or urine from humans and rats.

Food or supplement	Compounds	Metabolites	Parent mass	Fragments	Ref
Enterolignans					
Cocoa		Enterodiol	301	253	[14]
		Enterolactone	577	289	
Stilbenes					
Wine, resveratrol pill and standard	Resveratrol	*trans-/cis*-resveratrol	227	185	[69–71]
Wine, grape extract, resveratrol pill and standard		*trans-/cis*-resveratrol-glucuronide	403	227	[19,69–71]
		trans-/cis-resveratrol-sulfate	307	227	
Resveratrol pill		*trans*-resveratrol-glucuronide-sulfate	483	307	[71]
		trans-resveratrol-diglucuronide	579	403	
Wine		*trans-/cis*-piceid	389	227	[69]
		trans-/cis-piceid-glucuronide	565	389, 227	
		trans-/cis-piceid-sulfate	469	389, 227	
Wine, resveratrol pill		Dihydroresveratrol	229	123	[69,71]
		Dihydroresveratrol-glucuronide	405	229	
Wine, grape extract		Dihydroresveratrol-sulfate	309	229	[19,69]
Grape extract, resveratrol pill		Dihydroresveratrol-glucuronide sulfate	485	309/ 233/ 175	[19,71]
Ellagitannins					
Red raspberry	Ellagitannins	Urolithin A	227	182/171	[55]
		Urolithin A-glucuronide	403	175/171	
		Urolithin A-sulfate	307	175/113	
		Isourolithin A-glucuronide	403	171/175	
		(iso)Urolithin A-glucuronide-sulfate	483	403/277/307/175	
		Urolithin B	211	117/167	
		Urolithin B-glucuronide	387	175/113	
		Dimethylellagic acid-glucuronide	505	113/175	

Table S6. Metabolites of other phenolic compounds detected in plasma and/or urine from humans and rats.

Food or supplement	Compounds	Metabolites	Parent mass	Fragments	Ref
Wine, concord grape juice		Tartaric acid	149	/	[34,63]
Wine		Ethyl hydrogen-sulfate	125	/	[63]
		Monoglyceride citrate	265	/	
		Galactosylglycerol	253	/	
		Methylisocitric acid	187	/	
		Isopropyl-oxosuccinate	219	/	
		Dihydroxy-methoxyacetophenone-glucoside	343	/	
		Ethyl maltol	139	/	
		Phloroglucinol	125	/	[42,63]
		α-terpinyl cinnamate	283	/	[63]
		Licarin C/kanzonol R	369	/	
		Kanzonol I	435	/	
Grape extract, concord grape juice		Pyrogallol	125	97	[19,34]
Black tea, cranberry juice, beans, puree of (poly)phenol-rich berry fruits	Pyrogallol	Pyrogallol-sulfate	204	125	[29,30,38,41]
Cranberry juice, grape pomace drink, beans, puree of (poly)phenol-rich berry fruits		Methylpyrogallol-sulfate	218	139, 124	[29]
Black tea, puree of (poly)phenol-rich berry fruits	Pyrogallol	Pyrogallol-glucuronide	301	125	[29,30]
Black tea		Dihydroxyphenyl-sulfate	209	129	[72]
Grape extract		Methylpyrogallol	139	124	[29]
Cranberry (juice and extract), grape pomace drink, beans, puree of (poly)phenol-rich berry fruits, apple		Catechol-sulfate	189	109	[27,29,31,38,41,57]
Puree of (poly)phenol-rich berry fruits		Catechol-glucuronide	285		[29]
Cranberry juice, grape pomace drink, beans, puree of (poly)phenol-rich berry fruits, apple		Methylcatechol-sulfate	203	123	[27,29,38,41,57]
Puree of (poly)phenol-rich berry fruits		Methylcatechol-glucuronide	299		[29]
Black tea	Resorcinol	Diphenol-glucuronide	285	/	[30]
		Diphenol-sulfate	188	/	
Cranberry juice, beans		Hydroxybenzaldehyde	121	/	[38,41]
Cranberry juice		Dihydroxybenzaldehyde	137	/	[38]
EVOO, almond		Vanillin	151	136	[6,26]
EVOO		Vanillin-sulfate	231	151/136	[6]
		Vanillin-glucuronide	327		
Cranberry syrup		Scopoletin	193	/	[32]
		Methyl-scopoletin	207	/	
		Dihydroxycoumarin-glucuronide	355	/	
		Methyl-hydroxycoumarin-glucuronide	353	/	

References

[1] M.-C. López de las Hazas, C. Piñol, A. Macià, M.-P. Romero, A. Pedret, R. Solà, L. Rubió, M.-J. Motilva, Differential absorption and metabolism of hydroxytyrosol and its precursors oleuropein and secoiridoids, Journal of Functional Foods. 22 (2016) 52–63. https://doi.org/10.1016/j.jff.2016.01.030.

[2] M.I. Orozco-Solano, C. Ferreiro-Vera, F. Priego-Capote, M.D. Luque de Castro, Automated method for determination of olive oil phenols and metabolites in human plasma and application in intervention studies, Journal of Chromatography A. 1258 (2012) 108–116. https://doi.org/10.1016/j.chroma.2012.08.057.

[3] F.N. Bazoti, E. Gikas, A. Tsarbopoulos, Simultaneous quantification of oleuropein and its metabolites in rat plasma by liquid chromatography electrospray ionization tandem mass spectrometry, Biomed. Chromatogr. 24 (2010) 506–515. https://doi.org/10.1002/bmc.1319.

[4] L. Rubió, R.-M. Valls, A. Macià, A. Pedret, M. Giralt, M.-P. Romero, R. de la Torre, M.-I. Covas, R. Solà, M.-J. Motilva, Impact of olive oil phenolic concentration on human plasmatic phenolic metabolites, Food Chemistry. 135 (2012) 2922–2929. https://doi.org/10.1016/j.foodchem.2012.07.085.

[5] L. Rubió, A. Macià, R.M. Valls, A. Pedret, M.-P. Romero, R. Solà, M.-J. Motilva, A new hydroxytyrosol metabolite identified in human plasma: Hydroxytyrosol acetate sulphate, Food Chemistry. 134 (2012) 1132–1136. https://doi.org/10.1016/j.foodchem.2012.02.192.

[6] M. Suárez, R.M. Valls, M.-P. Romero, A. Macià, S. Fernández, M. Giralt, R. Solà, M.-J. Motilva, Bioavailability of phenols from a phenol-enriched olive oil, Br J Nutr. 106 (2011) 1691–1701. https://doi.org/10.1017/S0007114511002200.

[7] López-Yerena, Vallverdú-Queralt, Mols, Augustijns, Lamuela-Raventós, Escribano-Ferrer, Absorption and Intestinal Metabolic Profile of Oleocanthal in Rats, Pharmaceutics. 12 (2020) 134. https://doi.org/10.3390/pharmaceutics12020134.

[8] O. Khymenets, M.C. Crespo, O. Dangles, N. Rakotomanomana, C. Andres-Lacueva, F. Visioli, Human hydroxytyrosol's absorption and excretion from a nutraceutical, Journal of Functional Foods. 23 (2016) 278–282. https://doi.org/10.1016/j.jff.2016.02.046.

[9] O. Khymenets, M. Farré, M. Pujadas, E. Ortiz, J. Joglar, M.I. Covas, R. de la Torre, Direct analysis of glucuronidated metabolites of main olive oil phenols in human urine after dietary consumption of virgin olive oil, Food Chemistry. 126 (2011) 306–314. https://doi.org/10.1016/j.foodchem.2010.10.044.

[10] A. Serra, L. Rubió, A. Macià, R.-M. Valls, Ú. Catalán, Application of dried spot cards as a rapid sample treatment method for determining hydroxytyrosol metabolites in human urine samples. Comparison with microelution solid-phase extraction, (n.d.) 14.

[11] M. Suárez, M.-P. Romero, A. Macià, R.M. Valls, S. Fernández, R. Solà, M.-J. Motilva, Improved method for identifying and quantifying olive oil phenolic compounds and their metabolites in human plasma by microelution solid-phase extraction plate and liquid chromatography–tandem mass spectrometry, Journal of Chromatography B. 877 (2009) 4097–4106. https://doi.org/10.1016/j.jchromb.2009.10.025.

[12] M.-C. López de las Hazas, L. Rubió, A. Kotronoulas, R. de la Torre, R. Solà, M.-J. Motilva, Dose effect on the uptake and accumulation of hydroxytyrosol and its metabolites in target tissues in rats, Mol. Nutr. Food Res. 59 (2015) 1395–1399. https://doi.org/10.1002/mnfr.201500048.

[13] R. Domínguez-Perles, D. Auñón, F. Ferreres, A. Gil-Izquierdo, Gender differences in plasma and urine metabolites from Sprague–Dawley rats after oral administration of normal and high doses of hydroxytyrosol, hydroxytyrosol acetate, and DOPAC, Eur J Nutr. 56 (2017) 215–224. https://doi.org/10.1007/s00394-015-1071-2.

[14] M. Urpi-Sarda, M. Monagas, N. Khan, R.M. Lamuela-Raventos, C. Santos-Buelga, E. Sacanella, M. Castell, J. Permanyer, C. Andres-Lacueva, Epicatechin, procyanidins, and

phenolic microbial metabolites after cocoa intake in humans and rats, Anal Bioanal Chem. 394 (2009) 1545–1556. https://doi.org/10.1007/s00216-009-2676-1.
[15] M. Urpi-Sarda, M. Monagas, N. Khan, R. Llorach, R.M. Lamuela-Raventós, O. Jáuregui, R. Estruch, M. Izquierdo-Pulido, C. Andrés-Lacueva, Targeted metabolic profiling of phenolics in urine and plasma after regular consumption of cocoa by liquid chromatography–tandem mass spectrometry, Journal of Chromatography A. 1216 (2009) 7258–7267. https://doi.org/10.1016/j.chroma.2009.07.058.
[16] E. Roura, C. Andrés-Lacueva, O. Jáuregui, E. Badia, R. Estruch, M. Izquierdo-Pulido, R.M. Lamuela-Raventós, Rapid Liquid Chromatography Tandem Mass Spectrometry Assay To Quantify Plasma (−)-Epicatechin Metabolites after Ingestion of a Standard Portion of Cocoa Beverage in Humans, J. Agric. Food Chem. 53 (2005) 6190–6194. https://doi.org/10.1021/jf050377u.
[17] J.I. Ottaviani, T.Y. Momma, G.K. Kuhnle, C.L. Keen, H. Schroeter, Structurally related (−)-epicatechin metabolites in humans: Assessment using de novo chemically synthesized authentic standards, 52 (2012) 1403–1412. https://doi.org/10.1016/j.freeradbiomed.2011.12.010.
[18] Z. Shang, F. Wang, S. Dai, J. Lu, X. Wu, J. Zhang, Profiling and identification of (−)-epicatechin metabolites in rats using ultra-high performance liquid chromatography coupled with linear trap-Orbitrap mass spectrometer: Study of EC in vivo metabolites, Drug Test. Analysis. 9 (2017) 1224–1235. https://doi.org/10.1002/dta.2155.
[19] G. Sasot, M. Martínez-Huélamo, A. Vallverdú-Queralt, M. Mercader-Martí, R. Estruch, R.M. Lamuela-Raventós, Identification of phenolic metabolites in human urine after the intake of a functional food made from grape extract by a high resolution LTQ-Orbitrap-MS approach, Food Research International. 100 (2017) 435–444. https://doi.org/10.1016/j.foodres.2017.01.020.
[20] A. Serra, A. Macià, L. Rubió, N. Anglès, N. Ortega, J.R. Morelló, M.-P. Romero, M.-J. Motilva, Distribution of procyanidins and their metabolites in rat plasma and tissues in relation to ingestion of procyanidin-enriched or procyanidin-rich cocoa creams, Eur J Nutr. 52 (2013) 1029–1038. https://doi.org/10.1007/s00394-012-0409-2.
[21] A. Fardet, R. Llorach, J.-F. Martin, C. Besson, B. Lyan, E. Pujos-Guillot, A. Scalbert, A Liquid Chromatography−Quadrupole Time-of-Flight (LC−QTOF)-based Metabolomic Approach Reveals New Metabolic Effects of Catechin in Rats Fed High-Fat Diets, J. Proteome Res. 7 (2008) 2388–2398. https://doi.org/10.1021/pr800034h.
[22] M. Gómez-Juaristi, B. Sarria, S. Martínez-López, L. Bravo Clemente, R. Mateos, Flavanol Bioavailability in Two Cocoa Products with Different Phenolic Content. A Comparative Study in Humans, Nutrients. 11 (2019) 1441. https://doi.org/10.3390/nu11071441.
[23] E. Roura, C. Andrés-Lacueva, R. Estruch, M. Lourdes Mata Bilbao, M. Izquierdo-Pulido, R.M. Lamuela-Raventós, The effects of milk as a food matrix for polyphenols on the excretion profile of cocoa (−)-epicatechin metabolites in healthy human subjects, Br J Nutr. 100 (2008) 846–851. https://doi.org/10.1017/S0007114508922534.
[24] I. Hakeem Said, J.D. Truex, C. Heidorn, M.B. Retta, D.D. Petrov, S. Haka, N. Kuhnert, LC-MS/MS based molecular networking approach for the identification of cocoa phenolic metabolites in human urine, Food Research International. 132 (2020) 109119. https://doi.org/10.1016/j.foodres.2020.109119.
[25] L. Actis-Goretta, A. Lévèques, F. Giuffrida, F. Romanov-Michailidis, F. Viton, D. Barron, M. Duenas-Paton, S. Gonzalez-Manzano, C. Santos-Buelga, G. Williamson, F. Dionisi, Elucidation of (−)-epicatechin metabolites after ingestion of chocolate by healthy humans, Free Radical Biology and Medicine. 53 (2012) 787–795. https://doi.org/10.1016/j.freeradbiomed.2012.05.023.
[26] M. Urpi-Sarda, I. Garrido, M. Monagas, C. Gómez-Cordovés, A. Medina-Remón, C. Andres-Lacueva, B. Bartolomé, Profile of Plasma and Urine Metabolites after the Intake of Almond

[Prunus dulcis (Mill.) D.A. Webb] Polyphenols in Humans, J. Agric. Food Chem. 57 (2009) 10134–10142. https://doi.org/10.1021/jf901450z.
[27] F. Castello, G. Costabile, L. Bresciani, M. Tassotti, D. Naviglio, D. Luongo, P. Ciciola, M. Vitale, C. Vetrani, G. Galaverna, F. Brighenti, R. Giacco, D. Del Rio, P. Mena, Bioavailability and pharmacokinetic profile of grape pomace phenolic compounds in humans, Archives of Biochemistry and Biophysics. 646 (2018) 1–9. https://doi.org/10.1016/j.abb.2018.03.021.
[28] W. Mullen, G. Borges, M.E.J. Lean, S.A. Roberts, A. Crozier, Identification of Metabolites in Human Plasma and Urine after Consumption of a Polyphenol-Rich Juice Drink, J. Agric. Food Chem. 58 (2010) 2586–2595. https://doi.org/10.1021/jf904096v.
[29] R.C. Pimpão, T. Dew, M.E. Figueira, G.J. McDougall, D. Stewart, R.B. Ferreira, C.N. Santos, G. Williamson, Urinary metabolite profiling identifies novel colonic metabolites and conjugates of phenolics in healthy volunteers, Mol. Nutr. Food Res. 58 (2014) 1414–1425. https://doi.org/10.1002/mnfr.201300822.
[30] J. van Duynhoven, J.J.J. van der Hooft, F.A. van Dorsten, S. Peters, M. Foltz, V. Gomez-Roldan, J. Vervoort, R.C.H. de Vos, D.M. Jacobs, Rapid and Sustained Systemic Circulation of Conjugated Gut Microbial Catabolites after Single-Dose Black Tea Extract Consumption, J. Proteome Res. 13 (2014) 2668–2678. https://doi.org/10.1021/pr5001253.
[31] H. Liu, T.J. Garrett, F. Tayyari, L. Gu, Profiling the metabolome changes caused by cranberry procyanidins in plasma of female rats using ^{1}H NMR and UHPLC-Q-Orbitrap-HRMS global metabolomics approaches, Mol. Nutr. Food Res. 59 (2015) 2107–2118. https://doi.org/10.1002/mnfr.201500236.
[32] I. Iswaldi, D. Arráez-Román, A.M. Gómez-Caravaca, M. del M. Contreras, J. Uberos, A. Segura-Carretero, A. Fernández-Gutiérrez, Identification of polyphenols and their metabolites in human urine after cranberry-syrup consumption, Food and Chemical Toxicology. 55 (2013) 484–492. https://doi.org/10.1016/j.fct.2013.01.039.
[33] N. Brindani, P. Mena, L. Calani, I. Benzie, S.-W. Choi, F. Brighenti, F. Zanardi, C. Curti, D. Del Rio, Synthetic and analytical strategies for the quantification of phenyl-γ-valerolactone conjugated metabolites in human urine, Mol. Nutr. Food Res. 61 (2017) 1700077. https://doi.org/10.1002/mnfr.201700077.
[34] A. Stalmach, C.A. Edwards, J.D. Wightman, A. Crozier, Colonic catabolism of dietary phenolic and polyphenolic compounds from Concord grape juice, Food Function. 4 (2013) 52–62. https://doi.org/10.1039/C2FO30151B.
[35] C. Li, X. Meng, B. Winnik, M.-J. Lee, H. Lu, S. Sheng, B. Buckley, C.S. Yang, Analysis of Urinary Metabolites of Tea Catechins by Liquid Chromatography/Electrospray Ionization Mass Spectrometry, Chem. Res. Toxicol. 14 (2001) 702–707. https://doi.org/10.1021/tx0002536.
[36] K. Banaszewski, E. Park, I. Edirisinghe, J.C. Cappozzo, B.M. Burton-Freeman, A pilot study to investigate bioavailability of strawberry anthocyanins and characterize postprandial plasma polyphenols absorption patterns by Q-TOF LC/MS in humans, Journal of Berry Research. 3 (2013) 113–126. https://doi.org/10.3233/JBR-130048.
[37] X. Zeng, W. Su, Y. Bai, T. Chen, Z. Yan, J. Wang, M. Su, Y. Zheng, W. Peng, H. Yao, Urinary metabolite profiling of flavonoids in Chinese volunteers after consumption of orange juice by UFLC-Q-TOF-MS/MS, Journal of Chromatography B. 1061–1062 (2017) 79–88. https://doi.org/10.1016/j.jchromb.2017.07.015.
[38] R.P. Feliciano, E. Mecha, M.R. Bronze, A. Rodriguez-Mateos, Development and validation of a high-throughput micro solid-phase extraction method coupled with ultra-high-performance liquid chromatography-quadrupole time-of-flight mass spectrometry for rapid identification and quantification of phenolic metabolites in human plasma and urine, Journal of Chromatography A. 1464 (2016) 21–31. https://doi.org/10.1016/j.chroma.2016.08.027.

[39] A.K. Sandhu, Y. Huang, D. Xiao, E. Park, I. Edirisinghe, B. Burton-Freeman, Pharmacokinetic Characterization and Bioavailability of Strawberry Anthocyanins Relative to Meal Intake, J. Agric. Food Chem. 64 (2016) 4891–4899. https://doi.org/10.1021/acs.jafc.6b00805.
[40] A.K. Sandhu, M.G. Miller, N. Thangthaeng, T.M. Scott, B. Shukitt-Hale, I. Edirisinghe, B. Burton-Freeman, Metabolic fate of strawberry polyphenols after chronic intake in healthy older adults, Food Funct. 9 (2018) 96–106. https://doi.org/10.1039/C7FO01843F.
[41] E. Mecha, R.P. Feliciano, A. Rodriguez-Mateos, S.D. Silva, M.E. Figueira, M.C. Vaz Patto, M.R. Bronze, Human bioavailability of phenolic compounds found in common beans: the use of high-resolution MS to evaluate inter-individual variability, Br J Nutr. 123 (2020) 273–292. https://doi.org/10.1017/S0007114519002836.
[42] G. Pereira-Caro, J.L. Ordóñez, I. Ludwig, S. Gaillet, P. Mena, D. Del Rio, J.-M. Rouanet, K.A. Bindon, J.M. Moreno-Rojas, A. Crozier, Development and validation of an UHPLC-HRMS protocol for the analysis of flavan-3-ol metabolites and catabolites in urine, plasma and feces of rats fed a red wine proanthocyanidin extract, Food Chemistry. 252 (2018) 49–60. https://doi.org/10.1016/j.foodchem.2018.01.083.
[43] M. Martínez-Huélamo, S. Tulipani, X. Torrado, R. Estruch, R.M. Lamuela-Raventós, Validation of a New LC-MS/MS Method for the Detection and Quantification of Phenolic Metabolites from Tomato Sauce in Biological Samples, J. Agric. Food Chem. 60 (2012) 4542–4549. https://doi.org/10.1021/jf205266h.
[44] M. Wu, X. Wu, D. Zhang, F. Qiu, L. Ding, H. Ma, X. Chen, Metabolic profiling of quercetin in rats using ultra-performance liquid chromatography/quadrupole-time-of-flight mass spectrometry, Biomedical Chromatography. 31 (2017) e4016. https://doi.org/10.1002/bmc.4016.
[45] J. Lee, S.E. Ebeler, J.A. Zweigenbaum, A.E. Mitchell, UHPLC-(ESI)QTOF MS/MS Profiling of Quercetin Metabolites in Human Plasma Postconsumption of Applesauce Enriched with Apple Peel and Onion, J. Agric. Food Chem. 60 (2012) 8510–8520. https://doi.org/10.1021/jf302637t.
[46] M. Gómez-Juaristi, S. Martínez-López, B. Sarria, L. Bravo, R. Mateos, Absorption and metabolism of yerba mate phenolic compounds in humans, Food Chemistry. 240 (2018) 1028–1038. https://doi.org/10.1016/j.foodchem.2017.08.003.
[47] N. Hayasaka, N. Shimizu, T. Komoda, S. Mohri, T. Tsushida, T. Eitsuka, T. Miyazawa, K. Nakagawa, Absorption and Metabolism of Luteolin in Rats and Humans in Relation to in Vitro Anti-inflammatory Effects, J. Agric. Food Chem. 66 (2018) 11320-11329. https://doi.org/10.1021/acs.jafc.8b03273.
[48] W. Zhao, Z. Shang, Q. Li, M. Huang, W. He, Z. Wang, J. Zhang, Rapid Screening and Identification of Daidzein Metabolites in Rats Based on UHPLC-LTQ-Orbitrap Mass Spectrometry Coupled with Data-Mining Technologies, Molecules. 23 (2018) 151. https://doi.org/10.3390/molecules23010151.
[49] S. Saha, P.A. Kroon, A Simple and Rapid LC-MS/MS Method for Quantification of Total Daidzein, Genistein, and Equol in Human Urine, Journal of Analytical Methods in Chemistry. 2020 (2020) 1–9. https://doi.org/10.1155/2020/2359397.
[50] J. Zhang, Q. Guo, M. Wei, J. Bai, J. Huang, Y. Liu, Z. Su, X. Qiu, Metabolite Identification and Pharmacokinetic Profiling of Isoflavones from Black Soybean in Rats Using Ultrahigh-Performance Liquid Chromatography with Linear-Ion-Trap–Orbitrap and Triple-Quadrupole Tandem Mass Spectrometry, J. Agric. Food Chem. 66 (2018) 12941–12952. https://doi.org/10.1021/acs.jafc.8b04852.
[51] S. Tulipani, M. Martinez Huelamo, M. Rotches Ribalta, R. Estruch, E.E. Ferrer, C. Andres-Lacueva, M. Illan, R.M. Lamuela-Raventós, Oil matrix effects on plasma exposure and urinary excretion of phenolic compounds from tomato sauces: Evidence from a human pilot study, Food Chemistry. 130 (2012) 581–590. https://doi.org/10.1016/j.foodchem.2011.07.078.

[52] F. Castello, M.-S. Fernández-Pachón, I. Cerrillo, B. Escudero-López, Á. Ortega, A. Rosi, L. Bresciani, D. Del Rio, P. Mena, Absorption, metabolism, and excretion of orange juice (poly)phenols in humans: The effect of a controlled alcoholic fermentation, Archives of Biochemistry and Biophysics. 695 (2020) 108627. https://doi.org/10.1016/j.abb.2020.108627.
[53] Q. Jiao, L. Xu, L. Jiang, Y. Jiang, J. Zhang, B. Liu, Metabolism study of hesperetin and hesperidin in rats by UHPLC-LTQ-Orbitrap MS ^{n}, Xenobiotica. 50 (2020) 1311–1322. https://doi.org/10.1080/00498254.2019.1567956.
[54] C.D. Kay, G. Mazza, B.J. Holub, J. Wang, Anthocyanin metabolites in human urine and serum, Br J Nutr. 91 (2004) 933–942. https://doi.org/10.1079/BJN20041126.
[55] I.A. Ludwig, P. Mena, L. Calani, G. Borges, G. Pereira-Caro, L. Bresciani, D. Del Rio, M.E.J. Lean, A. Crozier, New insights into the bioavailability of red raspberry anthocyanins and ellagitannins, Free Radical Biology and Medicine. 89 (2015) 758–769. https://doi.org/10.1016/j.freeradbiomed.2015.10.400.
[56] D.N. Cooke, S. Thomasset, D.J. Boocock, M. Schwarz, P. Winterhalter, W.P. Steward, A.J. Gescher, T.H. Marczylo, Development of Analyses by High-Performance Liquid Chromatography and Liquid Chromatography/Tandem Mass Spectrometry of Bilberry (*Vaccinium myrtilus*) Anthocyanins in Human Plasma and Urine, J. Agric. Food Chem. 54 (2006) 7009–7013. https://doi.org/10.1021/jf061562q.
[57] S. Yuste, A. Macià, I.A. Ludwig, M.-P. Romero, S. Fernández-Castillejo, Ú. Catalán, M.-J. Motilva, L. Rubió, Validation of Dried Blood Spot Cards to Determine Apple Phenolic Metabolites in Human Blood and Plasma After an Acute Intake of Red-Fleshed Apple Snack, Mol. Nutr. Food Res. 62 (2018) 1800623. https://doi.org/10.1002/mnfr.201800623.
[58] M. Gómez-Juaristi, S. Martínez-López, B. Sarria, L. Bravo, R. Mateos, Bioavailability of hydroxycinnamates in an instant green/roasted coffee blend in humans. Identification of novel colonic metabolites, Food Funct. 9 (2018) 331–343. https://doi.org/10.1039/C7FO01553D.
[59] J.K. Aschoff, K.M. Riedl, J.L. Cooperstone, J. Högel, A. Bosy-Westphal, S.J. Schwartz, R. Carle, R.M. Schweiggert, Urinary excretion of Citrus flavanones and their major catabolites after consumption of fresh oranges and pasteurized orange juice: A randomized cross-over study, Mol. Nutr. Food Res. 60 (2016) 2602–2610. https://doi.org/10.1002/mnfr.201600315.
[60] C. Marmet, L. Actis-Goretta, M. Renouf, F. Giuffrida, Quantification of phenolic acids and their methylates, glucuronides, sulfates and lactones metabolites in human plasma by LC–MS/MS after oral ingestion of soluble coffee, Journal of Pharmaceutical and Biomedical Analysis. 88 (2014) 617–625. https://doi.org/10.1016/j.jpba.2013.10.009.
[61] M. Martínez-Huélamo, A. Vallverdú-Queralt, G. Di Lecce, P. Valderas-Martínez, S. Tulipani, O. Jáuregui, E. Escribano-Ferrer, R. Estruch, M. Illan, R.M. Lamuela-Raventós, Bioavailability of tomato polyphenols is enhanced by processing and fat addition: Evidence from a randomized feeding trial, Mol. Nutr. Food Res. 60 (2016) 1578–1589. https://doi.org/10.1002/mnfr.201500820.
[62] D. Scherbl, M. Renouf, C. Marmet, L. Poquet, I. Cristiani, S. Dahbane, S. Emady-Azar, J. Sauser, J. Galan, F. Dionisi, E. Richling, Breakfast consumption induces retarded release of chlorogenic acid metabolites in humans, Eur Food Res Technol. 243 (2017) 791–806. https://doi.org/10.1007/s00217-016-2793-y.
[63] A. Esteban-Fernández, C. Ibañez, C. Simó, B. Bartolomé, M.V. Moreno-Arribas, An Ultrahigh-Performance Liquid Chromatography–Time-of-Flight Mass Spectrometry Metabolomic Approach to Studying the Impact of Moderate Red-Wine Consumption on Urinary Metabolome, Journal of Proteome Research. 17 (2018) 1624–1635.
[64] Z. Zhang, M. Xu, S. Sun, X. Qiao, B. Wang, J. Han, D. Guo, Metabolic analysis of four phenolic acids in rat by liquid chromatography–tandem mass spectrometry, J. Chromatogr. B. 87 (2008) 7–14. https://doi.org/10.1016/j.jchromb.2008.06.019.

[65] A. Stalmach, W. Mullen, D. Barron, K. Uchida, T. Yokota, C. Cavin, H. Steiling, G. Williamson, A. Crozier, Metabolite Profiling of Hydroxycinnamate Derivatives in Plasma and Urine after the Ingestion of Coffee by Humans: Identification of Biomarkers of Coffee Consumption, Drug Metab Dispos. 37 (2009) 1749–1758. https://doi.org/10.1124/dmd.109.028019.
[66] G. Pereira-Caro, M.N. Clifford, T. Polyviou, I.A. Ludwig, H. Alfheeaid, J.M. Moreno-Rojas, A.L. Garcia, D. Malkova, A. Crozier, Plasma pharmacokinetics of (poly)phenol metabolites and catabolites after ingestion of orange juice by endurance trained men, Free Radical Biology and Medicine. 160 (2020) 784–795. https://doi.org/10.1016/j.freeradbiomed.2020.09.007.
[67] C. Favari, P. Mena, C. Curti, G. Istas, C. Heiss, D. Del Rio, A. Rodriguez-Mateos, Kinetic profile and urinary excretion of phenyl-γ-valerolactones upon consumption of cranberry: a dose–response relationship, Food Funct. 11 (2020) 3975–3985. https://doi.org/10.1039/D0FO00806K.
[68] A. Anesi, P. Mena, A. Bub, M. Ulaszewska, D. Del Rio, S.E. Kulling, F. Mattivi, Quantification of Urinary Phenyl-γ-Valerolactones and Related Valeric Acids in Human Urine on Consumption of Apples, Metabolites. 9 (2019) 254. https://doi.org/10.3390/metabo9110254.
[69] M. Rotches-Ribalta, C. Andres-Lacueva, R. Estruch, E. Escribano, M. Urpi-Sarda, Pharmacokinetics of resveratrol metabolic profile in healthy humans after moderate consumption of red wine and grape extract tablets, Pharmacological Research. 66 (2012) 375–382. https://doi.org/10.1016/j.phrs.2012.08.001.
[70] Z. Qiu, J. Yu, Y. Dai, X. Chen, F. Huang, N. Li, A simple LC–MS/MS method for the simultaneous quantification of resveratrol and its major phase II metabolites: Assessment of their urinary and biliary excretions in rats, Journal of Chromatography B. 1048 (2017) 85–93. https://doi.org/10.1016/j.jchromb.2017.02.011.
[71] L. Svilar, J.-C. Martin, C. Defoort, C. Paut, F. Tourniaire, A. Brochot, Quantification of trans-resveratrol and its metabolites in human plasma using ultra-high performance liquid chromatography tandem quadrupole-orbitrap mass spectrometry, Journal of Chromatography B. 1104 (2019) 119–129. https://doi.org/10.1016/j.jchromb.2018.11.016.
[72] C.A. Daykin, J.P.M.V. Duynhoven, A. Groenewegen, M. Dachtler, J.M.M.V. Amelsvoort, T.P.J. Mulder, Nuclear Magnetic Resonance Spectroscopic Based Studies of the Metabolism of Black Tea Polyphenols in Humans, J. Agric. Food Chem. 53 (2005) 1428–1434. https://doi.org/10.1021/jf048439o.
